# A multiantigenic Orf virus-based vaccine efficiently protects hamsters and nonhuman primates against SARS-CoV-2

**DOI:** 10.1038/s41541-024-00981-2

**Published:** 2024-10-16

**Authors:** Alena Reguzova, Melanie Müller, Felix Pagallies, Dominique Burri, Ferdinand Salomon, Hanns-Joachim Rziha, Zsofia Bittner-Schrader, Babs E. Verstrepen, Kinga P. Böszörményi, Ernst J. Verschoor, Ingo Gerhauser, Knut Elbers, Meral Esen, Alessandro Manenti, Martina Monti, Hans-Georg Rammensee, Madiha Derouazi, Markus W. Löffler, Ralf Amann

**Affiliations:** 1 Institute of Immunology, University Hospital Tübingen, Auf der Morgenstelle 15, 72076 Tübingen, Germany; 2 Speransa Therapeutics, Bethmannstrasse 8, 60311 Frankfurt am Main, Germany; 3 Department of Virology, Biomedical Primate Research Centre, Lange Kleiweg 161, 2288GJ Rijswijk, The Netherlands; 4 Department of Viroscience, Erasmus University Medical Center, Dr. Molewaterplein 50, 3015 GE Rotterdam, The Netherlands; 5 Department of Pathology, University of Veterinary Medicine Hannover Foundation, Bünteweg 17, 30559 Hannover, Germany; 6 Boehringer Ingelheim International GmbH, Binger Strasse 173, 55216 Ingelheim am Rhein, Germany; 7 ViraTherapeutics GmbH, Bundesstraße 27, 6063 Rum, Austria; 8 Institute of Tropical Medicine, University of Tübingen, Wilhelmstraße 27, 72074 Tübingen, Germany; 9 German Center for Infection Research (DZIF), Partner Site Tübingen; Cluster of Excellence (EXC2124) “Controlling Microbes to Fight Infection”, Tübingen, Germany; 10 VisMederi Srl., Strada del Petriccio e Belriguardo 35, 53100 Siena, Italy; 11 Cluster of Excellence iFIT (EXC2180) “Image-Guided and Functionally Instructed Tumor Therapies”, University of Tübingen, Tübingen, Germany; 12 Institute for Clinical and Experimental Transfusion Medicine, Medical Faculty of Tübingen, University Hospital Tübingen, Otfried-Müller-Str. 4/1, 72076 Tübingen, Germany; 13 Centre for Clinical Transfusion Medicine, Otfried-Müller-Str. 4/1, 72076 Tübingen, Germany

**Keywords:** Medical research, Preclinical research, Translational research, Drug development, Viral infection

## Abstract

Among the common strategies to design next-generation COVID-19 vaccines is broadening the antigenic repertoire thereby aiming to increase efficacy against emerging variants of concern (VoC). This study describes a new Orf virus-based vector (ORFV) platform to design a multiantigenic vaccine targeting SARS-CoV-2 spike and nucleocapsid antigens. Vaccine candidates were engineered, either expressing spike protein (ORFV-S) alone or co-expressing nucleocapsid protein (ORFV-S/N). Mono- and multiantigenic vaccines elicited comparable levels of spike-specific antibodies and virus neutralization in mice. Results from a SARS-CoV-2 challenge model in hamsters suggest cross-protective properties of the multiantigenic vaccine against VoC, indicating improved viral clearance with ORFV-S/N, as compared to equal doses of ORFV-S. In a nonhuman primate challenge model, vaccination with the ORFV-S/N vaccine resulted in long-term protection against SARS-CoV-2 infection. These results demonstrate the potential of the ORFV platform for prophylactic vaccination and represent a preclinical development program supporting first-in-man studies with the multiantigenic ORFV vaccine.

## Introduction

The COVID-19 pandemic has highlighted the need for vaccine platforms enabling rapid development and manufacturing of vaccines that provide long-term protection against severe disease.

While approved COVID-19 vaccines have demonstrated high protection against severe disease, their effectiveness against (re-)infection is limited^1^ and transient due to waning immunity over time^2^. Additionally, SARS-CoV-2 Variants of Concern (VoC) with constantly accumulating mutations represent a challenge for current vaccines^1^. To address this challenge, next-generation vaccines have been developed with the aim to stimulate broader humoral and cellular immunity by incorporating several immunodominant antigens^3–6^.

Addressing the well-conserved nucleocapsid protein as a promising target antigen in addition to the spike protein may improve vaccine efficacy^7–12^. Incorporating the nucleocapsid protein could substantially increase cell-mediated immunity^7^ and facilitate cross-reactive T-cell responses^13^. This strategy may enable protection independent of the constantly mutating spike protein^14^, thereby circumventing the challenge posed by future SARS-CoV-2 variants^8^. Currently, various approaches targeting nucleocapsid protein have been evaluated preclinically^15–22^ or are already in clinical development^23^ (ClinicalTrials.gov Identifiers: NCT04732468; NCT04546841; NCT04639466 and NCT04977024; NCT05370040).

Based on the Orf virus strain D1701-VrV (ORFV), a large (~140 kb) double-stranded DNA (dsDNA) virus from the genus *Parapoxvirus*, this study presents a novel vaccine platform. Designed to support multiantigenic vaccine concepts, this platform uniquely enables effective re-immunizations due to its characteristic of inducing only short-lived vector-specific immunity, while it can elicit strong and durable immune responses to vector-encoded antigens^24–31^. Unlike most viral vectors, ORFV can stably integrate multiple transgenes and is highly attenuated, not replicating in vivo^32^, which enhances its safety profile. Recently, a manufacturing process using HEK293F suspension cells was established for the production of vaccines under Good Manufacturing Practice conditions^33^. This process ensures the vaccine’s excellent stability, allowing for storage at +5 °C and at −20 °C for a minimum of 24 months^34^. These features make ORFV a promising candidate for the development of new vaccines.

Using the ORFV vector platform, we engineered COVID-19 vaccine candidates expressing either the stabilized full-length spike protein alone (ORFV-S, monoantigenic) or co-expressing nucleocapsid protein (ORFV-S/N, multiantigenic). We monitored humoral and cellular immunity in naïve mice, as well as in SARS-CoV-2 challenge models in hamsters and nonhuman primates (NHP). In mice, mono- and multiantigenic vaccines both induced comparable increases in anti-spike humoral and cellular immune responses. In hamsters, in contrast to the ORFV-S, vaccination with the ORFV-S/N vaccine conferred complete airway protection against ancestral SARS-CoV-2 (Wuhan). Modifying the spike antigen of the multiantigenic vaccine to the SARS-CoV-2 Beta VoC allowed for cross-protection against both the ancestral strain and the Delta VoC in hamsters. Moreover, we confirmed strong and long-lasting immunity along with protection against SARS-CoV-2 infection in NHPs vaccinated with the same multiantigenic ORFV-S/N vaccine. These results provide the basis for two phase 1 clinical studies assessing safety and dosing for a multiantigenic ORFV-based vaccine (ClinicalTrials.gov Identifiers: NCT05367843 and NCT05389319).

## Methods

### Study design

This study was designed to evaluate the immunogenicity and protective efficacy of ORFV-based vaccine candidates against SARS-CoV-2, containing the spike protein alone or in combination with nucleocapsid protein. Transgene expression was validated in cell culture by flow cytometry and Western blotting. Preclinical assessments were conducted in rodents and cynomolgus macaques. For in vitro and in vivo assays, sample sizes were calculated by the investigators on the basis of previous experience or suitable publications. All in vitro studies were performed at least twice with highly comparable outcomes.

### Ethical statement and experimental models

#### Mice

Male and female CD-1 mice were purchased and handled in strict accordance with the Federation of European Laboratory Animal Science Associations (FELASA) recommendations and followed the guidelines of the Regional councils. Experiments were carried out at the biosafety level 1 (BSL1) animal facility at the University of Tübingen, Germany, under the Project License Nr. IM1/20G.

#### Hamsters

Male Syrian golden hamsters were purchased, and all experiments were carried out under BSL3 conditions at Viroclinics Xplore animal facility (Schaijk, The Netherlands) under the Project License Nr. 27700202114492-WP02. All animals were treated in strict accordance with Dutch law for animal experimentation and Directive 2010/63/EU of the European Parliament and of the Council of September 22, 2010, on the protection of animals used for scientific purposes.

#### Macaques

Male and female rhesus macaques (*Macaca mulatta*) were mature, outbred animals, purpose-bred and housed at the Biomedical Primate Research Centre (BPRC) (Rijswijk, The Netherlands). Experiments were carried out under BSL3+ conditions under the Project License Nr. CCD 028H. The study was reviewed and approved by the Dutch “Centrale Commissie Dierproeven” (AVD5020020209404) according to Dutch law, article 10a of the “Wet op de Dierproeven” and BPRC’s Animal Welfare Body (IvD).

### Generation of D1701-VrV-based SARS-CoV-2 ORFV recombinants

The SARS-CoV-2 spike and nucleocapsid protein genes of the ancestral strain (Wuhan) or modified spike protein sequence containing three mutations (K417N, E484K, N501Y) in the RBD region characteristic for the SARS-CoV-2 Beta variant were synthesized by Gene Art (Thermo Fisher Scientific, Waltham, MA, USA). The spike or modified spike genes were cloned into plasmid pV12-Cherry; the nucleocapsid gene was cloned into plasmid pD12-Cherry^27^. Correct insertions and sequences were verified by restriction digestion and sequencing (Eurofins Genomics, Ebersberg, Germany). The resulting transfer plasmids were used for transfection of Vero cells (cat. no. CCL-81, American Type Culture Collection (ATCC), Manassas, VA, USA) infected with D1701-VrV-V12-Cherry using SF Cell Line 4D-Nucleofector^TM^ X Kit (Lonza, Köln, Germany) to replace the encoded Cherry gene by homologous recombination as described previously^27,35^. Transfer plasmids pV-CoV-Spike, pV-CoV-Spike-Beta and pD7-CoV-N were used to transfect Vero cells infected with D1701-VrV-GFP-D12-Cherry to replace GFP and Cherry genes, respectively. The new ORFV recombinants D1701-VrV-CoV-Spike, D1701-VrV-CoV-Spike-D7-CoV-N and D1701-VrV-CoV-Spike-Beta-D7-CoV-N were selected by fluorescence-activated cell sorting using a SH800S Cell Sorter (Sony Biotechnology, Bothell, WA, USA). Identification of ORFV recombinants was accomplished by polymerase chain reaction (PCR) using insert- and locus-specific primers. ORFV recombinants were propagated and purified as described previously^35^. Ten serial passages were performed in Vero cells to track the genetic stability of the inserted proteins. Virus titers were determined by a standard plaque assay^27^.

### In vitro transgene expression

Vero cells were seeded into 6-well plates (Greiner Bio-One, Frickenhausen, Germany) at a density of 5 × 10^5^ cells/well and infected with ORFV recombinants at a multiplicity of infection (MOI) of 1. For flow cytometry, protein expression was assessed 20 h post-infection. Cell surface staining was performed with SARS-CoV-2 Spike Neutralizing Antibody (cat. no. 40592-R001, Sino Biological, Eschborn, Germany) and anti-ORFV antibody (produced in-house), cells were fixed using Fixation & Permeabilization Solution (BD Biosciences, Franklin Lakes, NJ, USA) followed by intracellular staining with SARS-CoV-2 nucleocapsid antibody (cat. no. GTX135357, GeneTex, Irvine, CA, USA). Cells were acquired on a BD LSR Fortessa (BD Biosciences) and analyzed using the FlowJo^®^ v10 software (BD Biosciences). For Western blotting, supernatants obtained 48 h post-infection were collected, and cells were lysed in radioimmunoprecipitation assay (RIPA) lysis buffer containing protease/phosphatase inhibitors (Sigma-Aldrich, St. Louis, MO, USA). Cleared cell lysates were used for standard SDS-PAGE followed by immunoblot using SARS-CoV-2 spike neutralizing (anti-S1) (cat. no. 40592-R001, Sino Biological), spike (anti-S2) (cat. no. GTX632604, GeneTex), and nucleocapsid antibodies (cat. no. GTX135357, GeneTex). Anti-ORFV antibody (produced in-house) and anti-protein A antibody (Sigma-Aldrich) were used as an ORFV-infection and loading controls. Fusion FL camera and FusionCapt Advance software (PEQLAB, Erlangen, Germany) were used for membrane exposure. The uncropped and unprocessed western blot images are provided in Supplementary Fig. 15.

### Animal immunization and challenge experiments

#### Mice

CD-1 mice (Charles River Laboratories, Sulzfeld, Germany) were immunized intramuscularly (i.m.) at day 0 and 21 with ORFV-Mock, or ORFV-S or ORFV-S/N recombinants. For extended study, a third immunization was done on day 171. Alternatively, different ORFV-S/N doses were administered on days 0, 21 and 171, or on days 0 and 171 only. Peripheral blood was collected from the retro-orbital venous plexus under isoflurane (3–4%/O_2_) anesthesia on days 21, 28, 171 and 185. At the endpoint of experiments, mice were anesthetized with isoflurane and subsequently euthanized by cervical dislocation. Upon euthanasia, splenocytes were isolated utilizing standard procedures initially from five mice per group on day 28 and from the remainder of mice on day 185.

#### Hamsters

Syrian golden hamsters (Janvier, Le Genest-Saint-Isle, France) were immunized i.m. with ORFV-S/N, ORFV-S/N-Beta or ORFV-S recombinants at day 0 and 28 or with ORFV-S/N on day 28 only. PBS was injected at days 0 and 28 as a negative control. For comparison, hamsters were infected intranasally (i.n.) on day 0 with 10^2^ TCID_50_/dose of SARS-CoV-2 EU isolate BetaCoV/Munich/BavPat1/2020 (ancestral strain; Wuhan). Blood samples were collected on days 0, 28, 42 and 56. Animals were challenged with SARS-CoV-2 (ancestral strain; Wuhan or Delta variant strain) on day 56, administered as above. One, two and four days after the challenge, swabs of the respiratory tract were sampled. For all procedures, hamsters were sedated with isoflurane (3–4%/O_2_). At the endpoint of experiments, animals were anesthetized as stated above and euthanized by abdominal exsanguination, and tissue samples were obtained for analysis. For the study evaluating boosting effects of ORFV recombinants in SARS-CoV-2 experienced hamsters, animals were infected i.n. with SARS-CoV-2 (ancestral strain; Wuhan) as described above on day 0 and boosted with ORFV-S/N or ORFV-S recombinants on day 42. For comparison, hamsters were challenged with SARS-CoV-2 (ancestral strain; Wuhan) on day 42. Blood was collected on days 0, 21, 42, 49 and 56. For all procedures of this study, hamsters were sedated with isoflurane, as stated above.

#### Macaques

Rhesus macaques (*Macaca mulatta*) were immunized i.m. at day 0 and 28 with ORFV-S/N recombinant or PBS. Blood was obtained from the femoral vein in the groin on days 0, 14, 28, 42 and 70, and PBMCs on days 7 and 35. Macaques were challenged on day 70 with 10^5^ TCID_50_/dose of SARS-CoV-2 strain BetaCoV/German/BavPat1/2020 (ancestral strain; Wuhan-type) via a combined intranasal/intratracheal route. For the extended study, macaques were immunized i.m. at day 0 and 28 with different doses of ORFV-S/N recombinant or PBS. Serum was obtained on days 0, 14, 28, 42 and 147, and PBMCs on days 7 and 35. Animals were challenged on day 147, as described above. All macaques from both studies were monitored for SARS-CoV-2-related disease symptoms for the next 7–10 days. Tracheal and nasal swabs were collected daily after SARS-CoV-2 exposure, bronchoalveolar lavage (BAL) was obtained 3 days post-challenge. For all procedures, macaques were sedated with ketamine/medetomidine hydrochloride (10 mg/kg). When animals still showed muscle tension, they received additional medetomidine hydrochloride (0.05 mg/kg). After the virus inoculation Atipamezole hydrochloride (0.5 mg/kg) was used for faster recovery. Local anesthesia in the throat was given by spraying with 10% xylocaine. At the endpoint of experiments, all animals were euthanized for post-mortem tissue analysis. For that, macaques were first deeply sedated with ketamine/alfaxan and subsequently euthanized by intracardiac injection of a pentobarbiturate overdose.

### Detection of specific serum IgG

SARS-CoV-2-specific antibody titers were determined using SARS-CoV-2 S1 RBD- (IEQ-CoVS1RBD-IgG-5) and N-protein ELISA kit (IEQ-CoVN-IgG) (both Ray Biotech, Peachtree Corners, GA, USA) or SARS-CoV-2 Spike Trimer ELISA kit (Invitrogen, Carlsbad, CA, USA, BMS2324TEN). For detection of RBD(Beta)-specific antibody titers Nunc Maxisorp 96-well cell plates (Fisher Scientific, Schwerte, Germany) were coated with 1 µg/mL of recombinant RBD(Beta) protein (Sino Biological, 40592-V08H85) at 4 °C overnight followed by 2 h blocking using 1% BSA in PBS-Tween 0.05% at 37 °C. HRP-conjugated secondary antibodies were used to detect total bound IgG (1:5000, ab6728), IgG1 (1:1000, ab97240,) and IgG2a (1:1000, ab97245) subclasses in mouse serum, total bound IgG in hamster serum (1:10.000, ab6892) and in NHP serum (1:10.000, ab112767) (all Abcam, Cambridge, UK). All serum samples and controls were evaluated in duplicates using SpectraMax Plus 384 Microplate photometer (Molecular Devices, San Jose, California, USA). Blank absorbance units (OD) values were subtracted from the average values of sample duplicates. For calculation of the endpoint titer, the log10 OD values of the samples were plotted against the log10 of the sample dilution, and a regression analysis was performed. As a cut-off OD = 0.1 or the mean OD values at day 0 plus five times the standard deviation of day 0 OD values were used. Endpoint titer was defined as the value where the linear regression line of the cut-off intercepted with the regression line of the samples. WHO International Reference Panel with high levels of anti-SARS-CoV-2 immunoglobulin (NIBSC 20/150) was used as a standard.

### SARS-CoV-2 virus neutralization test

Analyses of samples from hamsters were done at Viroclinics Xplore (Schaijk, The Netherlands), NHP samples at Wageningen Bioveterinary Research (Lelystad, The Netherlands). Samples from mice were analyzed at Wageningen Bioveterinary Research (Lelystad, The Netherlands) or VisMederi Srl. (Siena, Italy). Mice samples were analyzed at VisMederi according to the protocol described in Manenti et al.^36^. Sera were heat-inactivated for 30 min at 56 °C. Sample dilutions in triplicates were incubated with 10^2^ TCID_50_/well of SARS-CoV-2 (ancestral strain; Wuhan) for 1 h at 37 °C (hamster) or in duplicates for 1.5 h at room temperature (macaques). Vero E6 cells (ATCC, CRL-1586) were added to the wells. Plates containing hamster serum were incubated for 5–6 days and scored for the presence of cytopathogenic effects (CPE) (100% endpoint) using a water-soluble tetrazolium salt (WST-8 cell proliferation assay; Promocell, Heidelberg, Germany). Virus neutralization titers (VNT) were calculated according to the method described by Reed and Muench^37^. For macaques’ samples, plates were incubated for 6–7 days. The virus-neutralizing titer was determined either microscopically or through an Immuno Peroxidase Monolayer Assay (IPMA) staining as the reciprocal dilution at which CPE reached 50%. WHO International Standard for anti-SARS-CoV-2 immunoglobulin (NIBSC 20/136) was used as a reference, where indicated.

### Intracellular cytokine staining (ICS)

Freshly isolated mouse splenocytes were seeded into a 96-well plate (Greiner Bio-One) at a density of 2 × 10^6^ cells/well and re-stimulated using 0.5 μg/mL of SARS-CoV-2 full-length spike- (PM-WCPV-S-1) or nucleocapsid peptide libraries (PM-WCPV-NCAP-1) (all JPT Peptide Technologies, Berlin, Germany) and 1 μg/mL of co-stimulatory anti-mouse CD28 (cat. no. 102116) and CD49d (cat. no. 103710) antibodies (all BioLegend, San Diego, CA, USA). After 1 h, 10 μg/mL of Brefeldin A (Sigma-Aldrich, St. Louis, MO, USA) was added for 14 h. Cells were first treated for 10 min with TruStain FcX™ (cat. no. 101320, BioLegend). Afterward, anti-mouse surface antibody cocktail was added for 30 min into the wells, containing CD3ε (cat. no. 100312), CD4 (cat. no. 100531), CD8a (cat. no. 100730), CD62L (cat. no. 104430) and CD44 (cat. no. 103022) antibodies in combination with Zombie Aqua Fixable viability dye (cat. no. 423102) (all BioLegend). Cells were further permeabilized for 30 min using Fixation & Permeabilization Solution (BD Bioscience) and incubated for 30 min with anti-mouse TNF (cat. no. 506324), IFN-γ (cat. no. 505835) and IL-2 (cat. no. 503808) antibody-mix (all BioLegend). Cells were acquired on a BD LSR Fortessa and analyzed using the FlowJo^®^ software. The background in unstimulated wells was subtracted from the peptide-stimulated wells.

### IFN-γ ELISpot assay

Cryopreserved PBMCs of macaques were thawed by standard procedure. Assays were performed in triplicates using Monkey IFN-γ ELISpot PLUS (ALP) kit (Mabtech, Stockholm, Sweden). Plates were blocked for 30 min with CTS OpTmizer medium (Gibco, Thermo Fisher Scientific) and 1 μg/mL of SARS-CoV-2 full-length spike- (PM-WCPV-S-1) or nucleocapsid peptide libraries (PM-WCPV-NCAP-1) (all JPT Peptide Technologies) in CTS OpTmizer medium was added into the wells. Next, 1–2 × 10^5^ cells were seeded into each well with *stimuli* and incubated for 20 h. Spots were counted on ImmunoSpot S6 Universal Analyzer CTL reader (Cellular Technology Limited, Shaker Heights, OH, USA). Mean of the triplicates was calculated and background in unstimulated wells was subtracted. Spot numbers were normalized to 1 × 10^6^ cells for each sample.

### Viral load in the respiratory tract

Detection of replication-competent SARS-CoV-2 virus in hamster nasal turbinate tissues, throat swabs and lungs post-challenge was performed at Viroclinics Xplore (Schaijk, The Netherlands). Briefly, sample serial dilutions were transferred in quadruplicates into 96-well plates (Greiner Bio-One), containing Vero E6 cell monolayers and incubated for 1 h at 37 °C. Afterward, cells were washed and incubated for 5 days. Plates were scored using WST-8 marker (Promocell) and viral titers (Log10 TCID_50_/mL or /g) were calculated using the Spearman-Karber’s method. Detection of viral RNA was performed by qPCR assay at Viroclinics Xplore (Schaijk, The Netherlands). Throat swabs and homogenized tissue samples were used to isolate RNA and Taqman PCR was performed using specific primers and probe. The number of copies (Log10 CP/mL) in the different samples was calculated against a standard included in each of the qPCR runs required.

Viral loads in the respiratory tract of challenged macaques were assessed at the Biomedical Primate Research Centre, the Netherlands. Briefly, SARS-CoV-2 genomic RNA was determined by real-time quantitative RT-PCR as described by Corman et al.^38^, a subgenomic (sgm) RT-qPCR was performed as described by Wölfel^39^ and Perera^40^. Viral RNA extractions were done using the QIAamp^®^ Viral RNA Mini Kit (Qiagen, Hilden, Germany). The cDNA synthesis and PCR amplification were performed using the Brilliant II qRT-PCR Core Reagent Kit, 1-Step kit (Agilent, Santa Clara, CA, USA), and RNA was quantified on a Bio-Rad CFX Connect real-time system (Bio-Rad Laboratories GmbH, Feldkirchen, Germany).

### Histopathology

Histopathological analysis of respiratory tract tissues was conducted for hamsters euthanized due to reaching experimental endpoint (day 60). After fixation with 10% formalin, tissue sections were embedded in paraffin and stained with hematoxylin and eosin (H&E). Histopathological assessment of stained slides was performed by a certified veterinary pathologist (I.G.) at the Department of Pathology, University of Veterinary Medicine (Hannover, Germany). Severity of rhinitis, bronchitis/bronchiolitis, and alveolitis was scored as follows: 0 = no inflammatory cells, 1 = few inflammatory cells, 2 = moderate numbers of inflammatory cells, 3 = many inflammatory cells. The extent of alveolitis was scored as follows: 0 = 0%; 1 ≤ 25%; 2 = 25–50%, 3 ≥ 50% of alveoli affected. The presence of alveolar edema, alveolar hemorrhage (≥2 erythrocytes in the lumen of one alveolus) and type II pneumocyte hyperplasia was determined. The extent of peribronchial/perivascular cuffing was scored as follows: 0 = none, 1 = 1–2 cells thick, 2 = 3–10 cells thick, 3 ≥ 10 cells thick.

Immunohistochemistry to detect CD3+ T cells was performed as described by Li et al.^41^. Briefly, after blocking of endogenous peroxidase activities (incubation with ethanol with 0.05% hydrogen peroxide for 30 min) and antigen retrieval (boiling in 10 mM citrate buffer, 20 min, pH 6.0) paraffin sections were blocked with PBS containing 20% goat serum for 30 min at room temperature. Sections were either incubated with a rabbit polyclonal antibody directed against CD3 (A0452, Agilent Technologies Germany GmbH & Co. KG, Waldbronn, Germany; 1:500) or rabbit serum as negative control (R4505, Merck KGaA, Darmstadt, Germany) overnight at 4 °C. Immunolabeling was visualized using a biotinylated goat-anti-rabbit antiserum (BA-1000, Vector Laboratories, Burlingame, CA, USA; 1:200), the avidin-biotin-peroxidase complex (ABC) method (PK-6100, Vector Laboratories), 3,3´-diaminobenzidine-tetrahydrochloride (DAB) as chromogen, and Mayer’s hematoxylin as counterstaining.

### Quantification and statistical analysis

Data were processed and analyzed with GraphPad Prism 9 software (GraphPad, San Diego, CA). Where applicable, the distributions of variables were assessed using the Shapiro–Wilk test of normality. Comparison among groups was performed by using the Mann–Whitney or Kruskal–Wallis test. *P*-values < 0.05 were considered statistically significant. Significance is annotated with respective symbols (*p < 0.05; **p < 0.01; ***p < 0.001). Data were presented as the means ± SEM (standard error of the mean) or as geometric mean with geometric standard deviation (SD), or as medians.

## Results

### Construction of ORFV D1701-VrV-based SARS-CoV-2 recombinants expressing spike protein alone or in combination with nucleocapsid protein

**Fig. 1:**
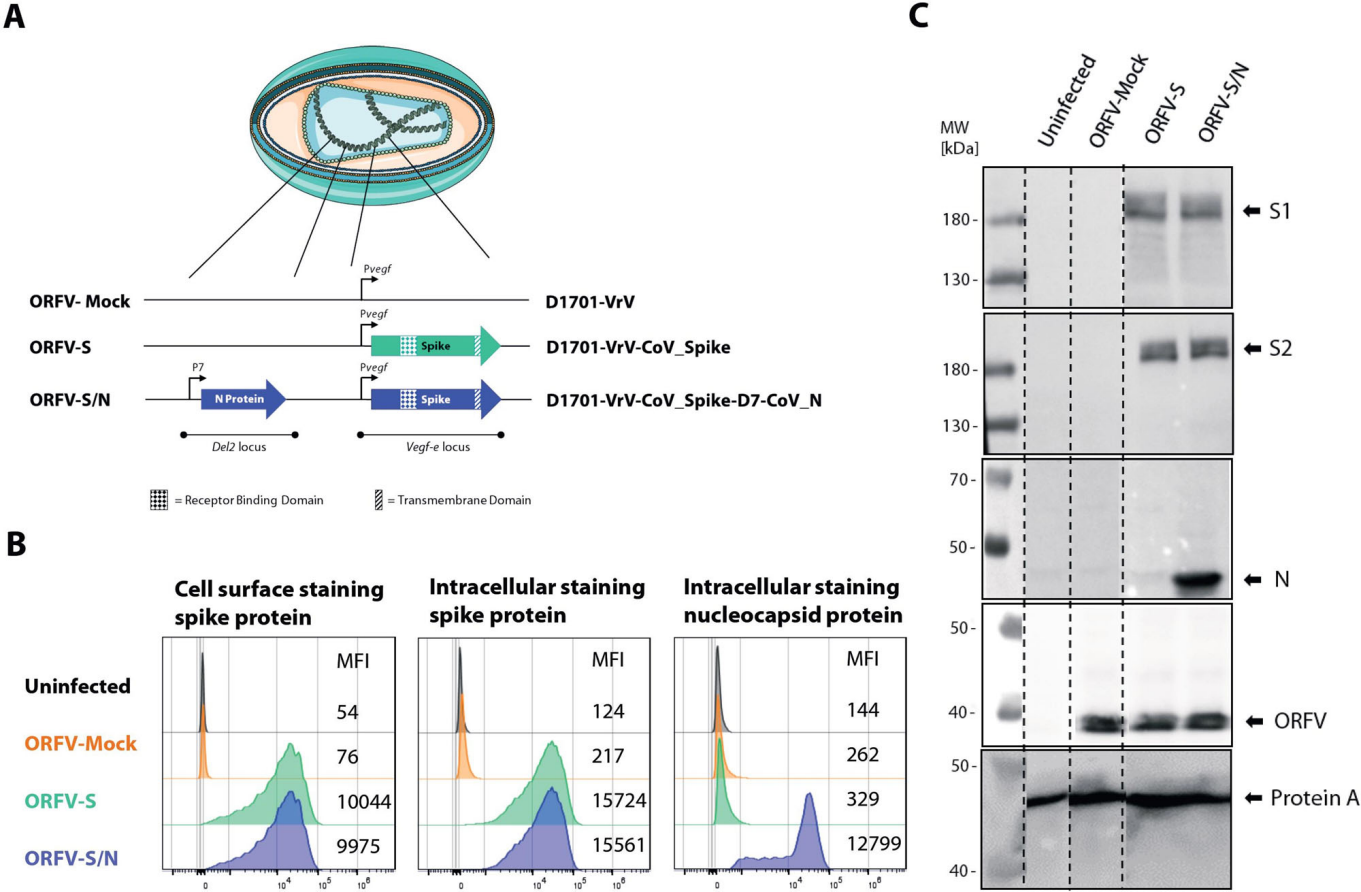
Construction and characterization of ORFV D1701-VrV-based SARS-CoV-2 recombinants. **A** Scheme of D1701-VrV (ORFV-Mock), and D1701-VrV-CoV_Spike (ORFV-S) and D1701-VrV-CoV_Spike-D7-CoV_N (ORFV-S/N) ORFV recombinants. Spike gene was inserted at the *vegf-e* locus and expressed under the control of the natural *Pvegf* promotor. Nucleocapsid gene was integrated into the D (*Del2)* locus under the control of an artificial *P7* promotor. **B** Flow cytometry analysis of Vero cells infected with the ORFV vectors for 20 h (MOI 1). Spike and nucleocapsid protein expression was evaluated on the cell surface and intracellularly using anti-S1, -S2, and -N antibodies, gating was done on ORFV-infected cells. Frequency of ORFV-infected cells was approximately 20% within each sample. Mean fluorescence intensity (MFI) is indicated for each sample. **C** Western Blot analysis of Vero cells infected (MOI 1) for 48 h with the ORFV-S and ORFV-S/N (cropped). Antigen expression was evaluated using anti-S1, -S2, -N and ORFV-antibodies in cell lysates. Broken lines indicate where the membrane was cut. Cells infected with ORFV-Mock or uninfected cells were used as controls as indicated. Created using images from Servier Medical Art (licensed under a Creative Commons Attribution 4.0 Unported License; https://creativecommons.org/licenses/by/4.0/).

We used the D1701-VrV ORFV vector^42,43^ to generate two SARS-CoV-2 vaccine candidates as described previously^27^ (Fig. 1A). The full-length spike protein from the genomic sequence of the ancestral SARS-CoV-2 (Wuhan)^44^ was inserted into the *vegf-e* locus under the control of the early *Pvegf* promotor of D1701-VrV ORFV in both recombinants. The spike protein sequence included mutations D614G, mutation K986P and V987P for proline stabilization and a furin cleavage site deletion (aa 682–685 RRAR to GSAS)^45^. The nucleocapsid gene was inserted in the D (*Del2)* locus under the control of an artificial early *P7* promotor, generating a double transgene-containing recombinant (ORFV-S/N) (Fig. 1A). The early *Pvegf* and P7 promotors initiated antigen expression encoded in the virus in the cytoplasm of the infected cells. We confirmed the integrity, correct and stable insertion of transgenes by vegf-, Del2- and transgene-specific PCR and gene sequencing. We used an empty D1701-VrV ORFV vector as a control (ORFV-Mock).

We confirmed spike protein expression from ORFV-S and ORFV-S/N by flow cytometry 20 h after infecting Vero cells using multiplicity of infection (MOI) of 1 by cell surface staining (Fig. 1B; left panel) and intracellular staining (Fig. 1B; middle panel). The mean fluorescence intensities were comparable for both recombinants. We also verified intracellular nucleocapsid protein production in Vero cells infected with the ORFV-S/N recombinant by flow cytometry (Fig. 1B; right panel) and confirmed the correct transgene expression by western blotting, using Vero cells harvested 48 h after infection (MOI 1) and detecting specific bands (180 kDa) in cell lysates using antibodies against S1 and S2 subunits of the spike protein (Fig. 1C), as well as against the nucleocapsid protein (46 kDa) (Fig. 1C).

### Mono- and multiantigenic ORFV recombinants induce robust and long-lasting T_H_1-biased antibody and cellular immune responses against inserted transgenes in mice

**Fig. 2:**
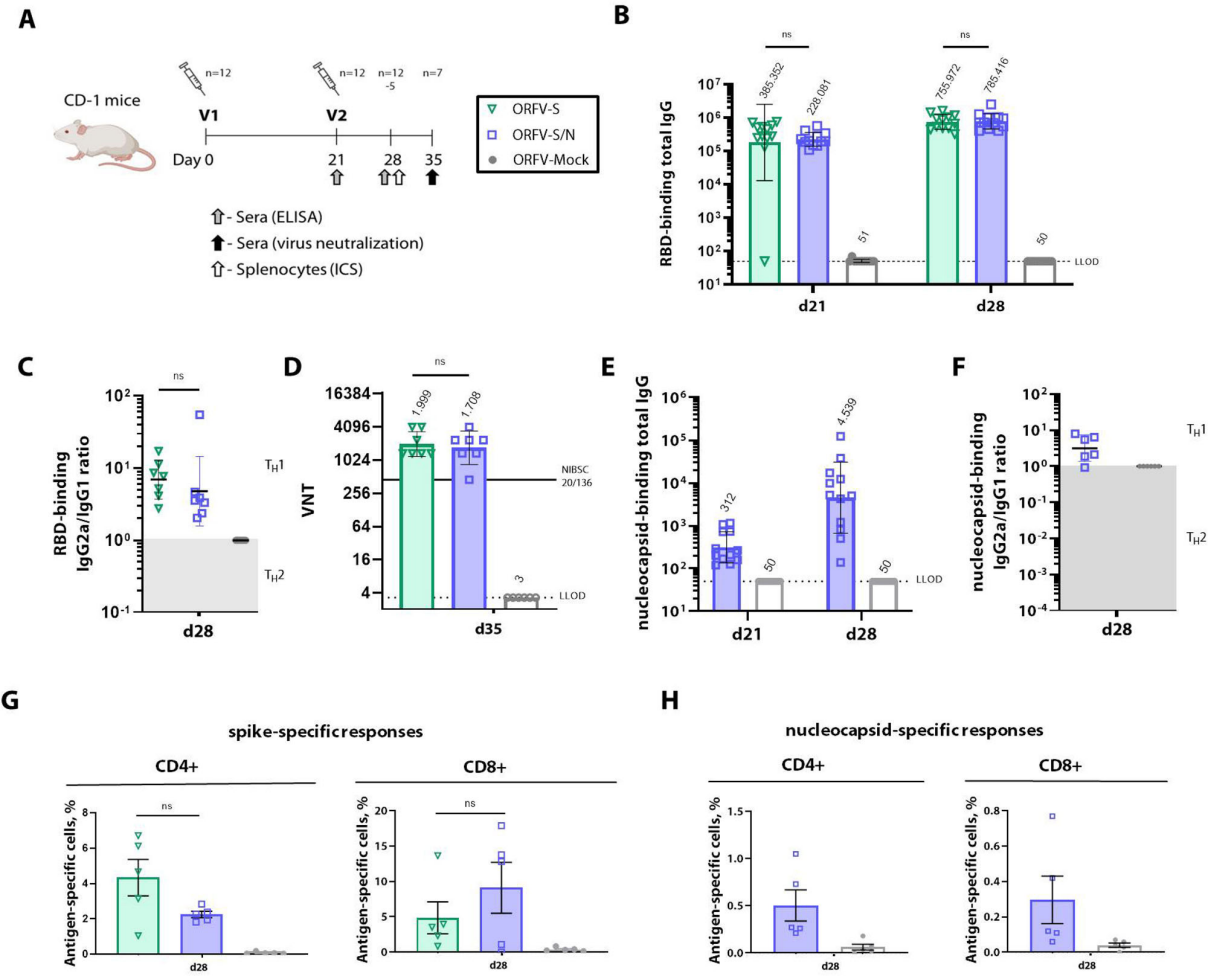
Immune responses stimulated by ORFV-S and ORFV-S/N recombinants in mice. **A** CD-1 mice were immunized at day 0 (V1) and 21 (V2) with 10^7^ PFU of ORFV-S and ORFV-S/N recombinants, or ORFV-Mock. **B** RBD-specific total IgG endpoint titers in mouse serum, evaluated 3 weeks after the first (day 21) and one/two weeks after the second (day 28/day 35) immunization by ELISA. **C** IgG2a/IgG1 isotype ratio of RBD-specific binding antibodies on day 28. **D** SARS-CoV-2 (ancestral strain; Wuhan) serum virus-neutralizing titer (VNT) on day 35. The horizontal solid line marks VNT in the WHO International Standard for anti-SARS-CoV-2 immunoglobulin NIBSC 20/136 reference panel. **E** Endpoint titers of nucleocapsid-specific total IgG in mouse serum after the first (day 21) and second (day 28) immunization by ELISA. **F** IgG2a/IgG1 isotype ratio of nucleocapsid-specific binding antibodies on day 28. In (**B**–**F**), data are presented as geometric mean values ± geometric standard deviation (SD). The dotted line indicates the lower limit of detection (LLOD). Geometric mean titers (GMT) are noted above the columns. **G** Total percentage of spike- and **H** nucleocapsid-specific CD4+ and CD8+ T cells producing IFNγ, TNF and IL-2 determined by flow cytometry. In (**G**) and (**H**), the heights of bars indicate mean ± SEM (standard error of the mean). Significance was assessed by the Kruskal–Wallis test. ns, not significant; *p < 0.05; **p < 0.01; ***p < 0.001. Images created in BioRender. Amann, R. (2024) BioRender.com/r59y162.

The immunogenicity of both ORFV-based vaccine candidates was evaluated in CD-1 mice after two intramuscular (i.m.) injections with a 21-day interval using 10^7^ plaque-forming units (PFU). Mice immunized with ORFV-Mock recombinant were used as controls (Fig. 2A). After a single injection, both ORFV vaccines induced similar levels of receptor binding domain (RBD)-specific binding antibodies with geometric mean endpoint titers (GMT) of 3.9 × 10^5^ and 2.3 × 10^5^, respectively, when assessed 3 weeks after vaccination (Fig. 2B). A second dose of either ORFV-S or ORFV-S/N increased the antigen-specific binding antibody titers at day 28 by 1.9- and 3.4-fold, respectively. The IgG2a/IgG1 isotype ratio of RBD-binding antibodies at day 28 showed a T_H_1-biased profile skewed toward IgG2a for both ORFV-S and ORFV-S/N (Fig. 2C). Virus-neutralizing titers (VNT) were similar for both constructs, with a GMT of 2.0 × 10^3^ and 1.7 × 10^3^ for ORFV-S and ORFV-S/N, respectively, compared to 4.5 × 10^2^ in the WHO International Standard of anti-SARS-CoV-2 immunoglobulin NIBSC 20/136 (Fig. 2D).

The ORFV-S/N also induced a robust antibody response to the nucleocapsid, which was increased by 14.5-fold after the second vaccination (Fig. 2E). The specific IgG2a/IgG1 ratios confirmed a T_H_1 profile after ORFV-S/N immunization (Fig. 2F).

In addition to the humoral immune response, both ORFV-S and ORFV-S/N recombinants elicited multifunctional CD4+ and CD8+ T cells specific for spike (Fig. 2G, Supplementary Fig. 1A, C) and nucleocapsid for the latter (Fig. 2H, Supplementary Fig. 1B, D), as monitored in spleens seven days after the second vaccination. Following two vaccinations with either ORFV-S or ORFV-S/N, the anti-spike immune responses comprised 4.3% and 2.3% of CD4+ T cells along with 4.8% and 9.1% of CD8+ T cells (Fig. 2G, Supplementary Fig. 1A). In ORFV-S/N vaccinated animals, frequencies of specific CD4+ and CD8 +T cells against nucleocapsid (0.5% and 0.3%, respectively) resulted considerably lower as compared to respective spike-directed T cells (Fig. 2H, Supplementary Fig. 1B).

The long-term persistence of the vaccine-induced humoral responses, as well as boost capacity of the ORFV-based SARS-CoV-2 vaccine candidates, was further assessed in mice (Supplementary Fig. 2A). ORFV-S and ORFV-S/N recombinants elicited elevated RBD-binding total IgG levels over the prolonged follow-up period, and a third vaccination (day 171) boosted pre-existing antibody levels by 2.2- and 1.5-fold for ORFV-S and ORFV-S/N, respectively (Supplementary Fig. 2B). The antibody levels against nucleocapsid were boosted by 44.6-fold following the third vaccination using ORFV-S/N (Supplementary Fig. 2C). VNT after the second (day 35) and the third vaccination (day 185) for both ORFV-S and ORFV-S/N were similar (Supplementary Fig. 2D). A third vaccination with ORFV-S or ORFV-S/N after a follow-up period of 150 days increased VNT by 1.7- and 3.0-fold, respectively, when determined by a protocol described in Manenti et al.^36^.

### Multiantigenic ORFV-S/N recombinant confers protection to vaccinated Syrian hamsters following SARS-CoV-2 challenge as compared to the monoantigenic ORFV-S

**Fig. 3:**
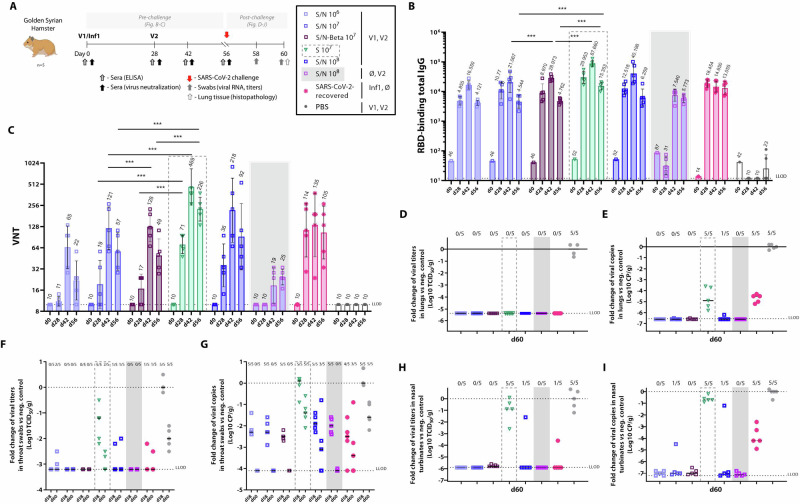
Protection against SARS-CoV-2 by vaccination with ORFV-S and ORFV-S/N recombinants in Syrian hamsters. **A** Hamsters were immunized with ORFV-S/N doses ranging from 10^6^ to 10^8^ PFU at days 0 (V1) and 28 (V2), or at day 28 only (V2) with 10^8^ PFU, or with 10^7^ PFU of ORFV-S for comparison. Two doses (10^7^ PFU) of multiantigenic SARS-CoV-2 vaccine candidate containing mutations in the spike protein (K417N, E484K, N501Y) characteristic for the SARS-CoV-2 Beta VoC (ORFV-S/N-Beta) were injected at days 0 (V1) and 28 (V2). PBS was administered as negative control. As a reference, hamsters were inoculated intranasally with 10^2^ TCID_50_ SARS-CoV-2 (ancestral strain; Wuhan) on day 0 (Inf1) (SARS-CoV-2-recovered). All animals were challenged with 10^2^ TCID_50_ SARS-CoV-2 (ancestral strain; Wuhan) at day 56 and monitored for 4 days. **B** Endpoint titers of RBD-specific total IgG in serum. **C** VNT of SARS-CoV-2 (ancestral strain; Wuhan) in serum. In (**B**) and (**C**), data are presented as geometric mean values ± geometric standard deviation (SD). GMT are noted above the columns. Fold change of SARS-CoV-2 viral **D** infectious titers and **E** RNA copies in lungs at day 60, **F** infectious titers and **G** RNA copies in throat swabs at days 58 and 60, and **H** infectious titers and **I** RNA copies in nasal turbinates at day 60, normalized to PBS group. In (**D**–**I**), data are presented as medians. Ratios above bars indicate the number of virus-positive animals per group. The dotted line indicates the lower limit of detection (LLOD). Significance was assessed by the Kruskal–Wallis test. *p < 0.05; **p < 0.01; ***p < 0.001. Images created in BioRender. Amann, R. (2024) BioRender.com/d60i449.

The Syrian hamster SARS-CoV-2 challenge model is a well-established system to study severe COVID-19 with pathology similar to humans^46^. In this study, hamsters were vaccinated twice at a 4-week interval with either escalating doses (10^6^–10^8^ PFU) or once with the highest dose (10^8^ PFU) of the ORFV-S/N recombinant, or two doses (10^7^ PFU) of ORFV-S recombinant or PBS. Besides, two doses (10^7^ PFU) of a multiantigenic SARS-CoV-2 vaccine candidate containing mutations in the RBD of the spike protein (K417N, E484K, N501Y) characteristic for the SARS-CoV-2 Beta VoC (ORFV-S/N-Beta) were administered. In addition, a group of hamsters was infected with SARS-CoV-2 (10^2^ TCID_50_ intranasally) (ancestral strain; Wuhan) at day 0 (SARS-CoV-2-recovered). All animals were challenged with SARS-CoV-2 (10^2^ TCID_50_) (ancestral strain; Wuhan) at day 56 (Fig. 3A).

After the first and second vaccinations, a dose-dependent increase of RBD-binding IgG antibodies and VNT (vs. ancestral strain) was observed in all groups (Fig. 3B, C). A correlation was also observed between total antibody levels and VNT. Notably, in contrast to mice, two doses of ORFV-S resulted in significantly higher RBD-binding antibody levels and VNT compared to equal doses of ORFV-S/N and ORFV-S/N-Beta (Fig. 3B, C).

Four days after the SARS-CoV-2 challenge, viral loads quantified for viral RNA and infectious titers were assessed in the nose, throat and lungs of hamsters, along with lung histopathology. Animals vaccinated with ORFV-S/N and ORFV-S/N-Beta were negative regarding infectious titers and viral loads in the lungs, while ORFV-S-vaccinated hamsters and those recovered from a previous SARS-CoV-2 infection showed reduced viral RNA loads in the lungs (Fig. 3D, E). All vaccinated animal groups exhibited lower viral loads and infectious titers in throat (Fig. 3F, G) and nose (Fig. 3H, I) as compared to the PBS controls. However, only the ORFV-S/N vaccine consistently reduced or prevented the presence of infectious virus titers in the upper respiratory tracts, despite lower antibody and VNT (Fig. 3F–I). Similarly, ORFV-S/N-Beta conferred cross-protection against the ancestral SARS-CoV-2 strain (Fig. 3D–I).

Histopathological analysis revealed that ORFV-S/N vaccination conferred protection against SARS-CoV-2, causing minimal affection of the upper and lower airways (Supplementary Table 1). All ORFV-S/N and ORFV-S/N-Beta vaccinated hamsters exhibited mild rhinitis with few lymphocytes, plasma cells, macrophages and neutrophils in the nasal mucosa (Supplementary Fig. 3). Severity scores ranged from 0.8 to 1.2 (Supplementary Table 1). Conversely, ORFV-S vaccinated, SARS-CoV-2-recovered and placebo-treated groups had more severe nasal lesions, with scores of 2.8, 2.0 and 3.0, respectively. These were characterized by a high presence of leukocytes in the nasal mucosa, accumulations of neutrophils within the lumen and shedding of epithelial cells. Inflammatory changes in the trachea were observed in 8 out of 20 animals vaccinated with ORFV-S/N, 4 out of 5 animals immunized with ORFV-S, and in all 5 animals from the SARS-CoV-2-recovered or PBS groups. These tracheal lesions were slightly less severe than those in the nasal cavity (Supplementary Table 1). The severity of tracheitis was most pronounced in the PBS group, whereas it was significantly diminished or not present in animals that received vaccinations (Supplementary Fig. 4). Lesions in the pulmonary parenchyma were generally mild or even absent in ORFV-S/N, ORFV-S/N-Beta and ORFV-S vaccinated animals (Supplementary Table 1, Supplementary Fig. 5). These lesions were characterized by increased numbers of histiocytic cells in alveolar lumina (alveolar histiocytosis), which were interspersed with a low number of neutrophils in single hamsters. Animals recovered from a previous SARS-CoV-2 infection or in the PBS group were affected by alveolitis resulting in higher scores (Supplementary Table 1, Supplementary Fig. 6). Few PBS control hamsters showed vascular lesions/endothelialitis. Inflammatory changes in the bronchi and bronchioles characterized by mucosal infiltrates with lymphocytes, plasma cells, macrophages and neutrophils were only found in PBS control animals (Supplementary Fig. 5) and were often associated with vascular lesions. Moreover, two ORFV-S/N-Beta vaccinated hamsters and three control animals were affected by mild or mild to moderate perivascular and peribronchial cuffing, respectively (Supplementary Table 1). Alveolar edema and type II pneumocyte hyperplasia were not found in hamsters. However, mild focal intraalveolar hemorrhages (few erythrocytes in the lumina of up to 10 alveoli) were noted in one animal vaccinated twice with ORFV-S, one animal from the SARS-CoV-2-recovered group, three animals from the PBS control group, and two animals vaccinated with a high dose (10^8^ PFU) of the ORFV-S/N (Supplementary Table 1, Supplementary Fig. 7). The macroscopic examination was restricted to the lung and revealed that none of the ORFV-S/N and ORFV-S/N-Beta vaccinated animals showed affected lung tissue except for one animal showing slightly (5%) affected tissue (Supplementary Table 2). In contrast, the lungs of 4 out of 5 animals in the SARS-CoV-2-recovered group and all animals in the ORFV-S or PBS control groups displayed more extensive damage, with 10–90% of the tissue affected. Immunohistochemistry staining for CD3+ cells showed minimal presence of CD3+ cells in nasal and tracheal mucosa vaccinated with ORFV-S/N, in contrast to higher levels observed in the ORFV-S vaccinated, SARS-CoV-2-recovered and PBS control groups (Supplementary Fig. 8). This suggests a limited infiltration of T cells. Moreover, the tracheal mucosa of hamsters vaccinated with ORF-S/N as well as those vaccinated with ORFV-S and those recovered from SARS-CoV-2 displayed significantly fewer CD3+ cells compared to the control animals (Supplementary Fig. 9). Similarly, lung immunohistochemistry findings showed that the PBS control group had increased CD3+ cell counts, associated with bronchiolitis and alveolitis, conditions not observed in the vaccinated or recovered groups (Supplementary Fig. 10). In summary, the histopathological (macro- and microscopic) examination of nasal cavity, trachea and lungs after challenge showed that immunization with ORFV-S, ORFV-S/N and ORFV-S/N-Beta vaccine candidates did not appear to result in inflammatory changes associated with immunopathology following vaccination, whereas endothelialitis with T cells infiltrating pulmonary blood vessels was found in the PBS control group.

Correlates of protection analyses showed that high VNT elicited by the ORFV-S recombinant did not cluster with reduced viral titers in nasal turbinates, in contrast to the effects observed after one or two administrations of ORFV-S/N or ORFV-S/N-Beta. Multiantigenic vaccines elicited better protection against viral challenge that included the upper airways (Supplementary Fig. 11A). In addition, no correlation was observed between the VNT after vaccination with ORFV-S, ORFV-S/N and ORFV-S/N-Beta recombinants and the elevated virus titers in lungs after the challenge (Supplementary Fig. 11B). Clustering of higher VNT levels with reduced histopathology scores in ORFV-S/N vaccinated animals suggested possible vaccine dose-dependent effects (Supplementary Fig. 11C).

Vaccine-induced cross-protection against the SARS-CoV-2 Delta variant was further demonstrated in a hamster challenge model (Supplementary Fig. 12). Therefore, two vaccinations of ORFV-S/N-Beta (3 × 10^7^ PFU) were administered, which fully protected the lungs and reduced viral loads of replication-competent SARS-CoV-2 Delta virus in the throat (1.6-fold) and nasal turbinates (2.4-fold) on days 1–4 post-challenge, as compared to PBS control animals. The severity scores concerning tracheitis, bronchiolitis, and alveolitis, along with the extent of alveolar inflammation, edema and hemorrhage, and the degree of peribronchial/perivascular cuffing, were lower in vaccinated hamsters.

The potential of both ORFV-S and ORFV-S/N vaccine candidates to boost pre-existing memory responses after SARS-CoV-2 infection was also investigated in the hamster model. Animals were infected with SARS-CoV-2 (10^2^ TCID_50_ intranasally) (SARS-CoV-2-recovered) and 42 days later either vaccinated with 3 × 10^7^ PFU of ORFV-S and ORFV-S/N, or re-infected with SARS-CoV-2. Hamsters were monitored until day 56 (Supplementary Fig. 13A). Already seven days after the boost with either ORFV-S or ORFV-S/N (day 49), increased RBD-binding IgG titers were observed (Supplementary Fig. 13B; Supplementary Table 3). Administration of ORFV-S or ORFV-S/N resulted in 4.5- or 2.6-fold higher RBD-specific antibody levels when compared to re-infection, respectively. Additionally, both vaccinations with the ORFV-S/N recombinant as well as the SARS-CoV-2 re-challenge boosted nucleocapsid-specific antibody levels (Supplementary Fig. 13C; Supplementary Table 4). Finally, administration of ORFV-S or ORFV-S/N after SARS-CoV-2 infection increased VNT by 3.6- and 3.5-fold as compared to re-infection, respectively (Supplementary Fig. 13D; Supplementary Table 5).

### Multiantigenic ORFV-S/N recombinant protects vaccinated nonhuman primates from SARS-CoV-2 infection

**Fig. 4:**
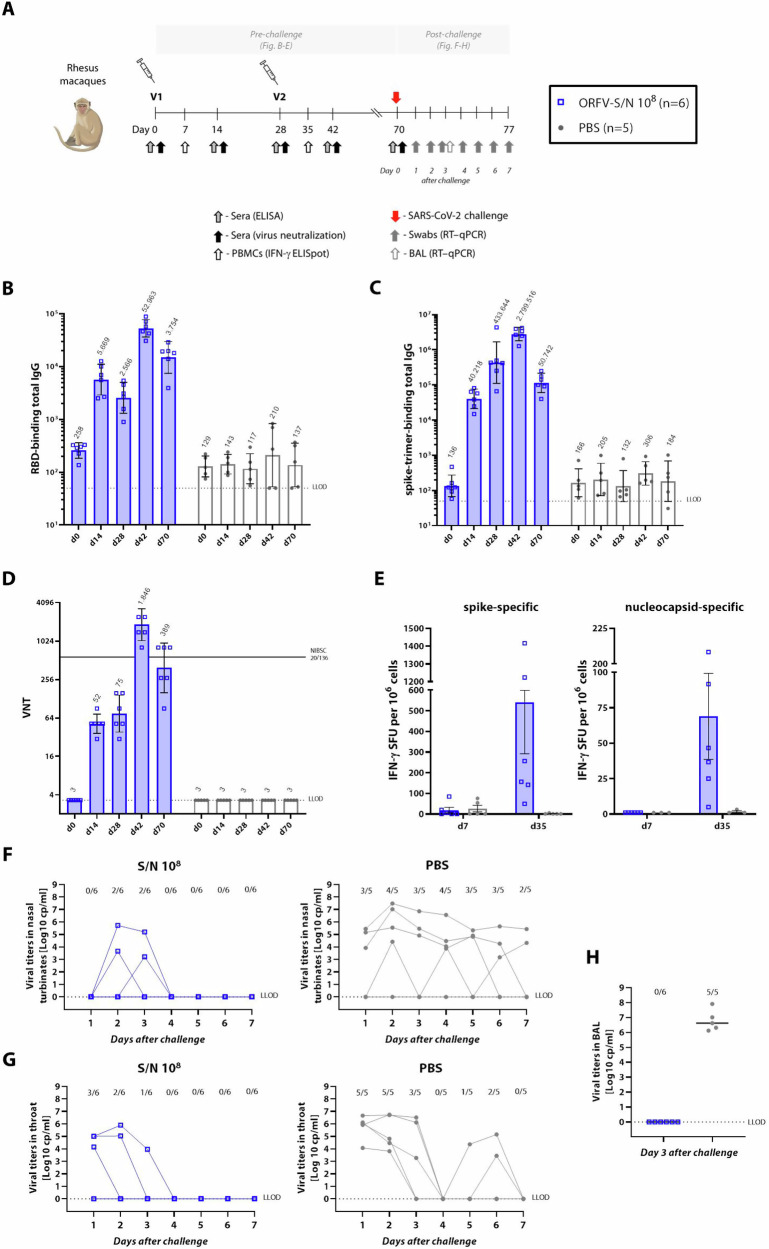
ORFV-S/N vaccine-induced protection in nonhuman primates (NHP). **A** NHPs were immunized twice with 10^8^ PFU of ORFV-S/N recombinant or PBS at days 0 and 28. All animals were challenged with 10^5^ TCID_50_ SARS-CoV-2, administered into nose and trachea at day 70 and monitored for the following seven days. Endpoint titers of **B** RBD- and **C** spike-trimer-specific total IgG in serum analyzed by ELISA. **D** SARS-CoV-2 (ancestral strain; Wuhan) VNT in serum. Solid line indicates VNT in NIBSC 20/136 reference panel. In (**B**–**D**), data are presented as geometric mean values ± geometric standard deviation (SD). GMT are noted above the columns. **E** IFNγ-secreting cells in PBMCs at day 7 and day 35 after 20 h stimulation with overlapping spike (left graph) and nucleocapsid (right graph) peptide pools, determined by ELISpot. In (**E**), the heights of bars indicate mean ± SEM (standard error of the mean). SARS-CoV-2 viral loads in **F** nose, **G** throat and **H** bronchoalveolar lavage (BAL) at indicated time points after challenge. In (**F**–**H**), data are presented as medians. Ratios above bars denote the number of viral RNA-positive macaques per group. The dotted line indicates the lower limit of detection (LLOD). Images created in BioRender. Amann, R. (2024) BioRender.com/a19n200.

The superior protection observed in the Syrian hamster model following the SARS-CoV-2 challenge prompted an evaluation of ORFV-S/N in an NHP challenge model. NHPs received either two doses of 10^8^ PFU of the ORFV-S/N recombinant with a 4-week interval or PBS as a control (Fig. 4A). Serum and peripheral blood mononuclear cells (PBMCs) were sampled regularly, and animals were challenged with 10^5^ TCID_50_ SARS-CoV-2, administered into nose and trachea on day 70. Subsequently, SARS-CoV-2 subgenomic messenger RNA (CoVsg) levels were determined daily in nose and throat over 1 week, and viral titers in bronchoalveolar lavage (BAL) were assessed on day 3 following the challenge.

RBD-binding IgG became detectable within 14 days after the first vaccination reaching GMT of 5.0 × 10^4^ (Fig. 4B). The second vaccination boosted antibody titers by 9-fold 2 weeks post-vaccination. Levels of spike-trimer-binding total IgG and VNT exhibited similar response patterns (Fig. 4C, D). Before challenge, RBD-binding IgG, spike-trimer-binding IgG and neutralizing GMT accounted for 1.4 × 10^4^, 5.0 × 10^4^ and 3.4 × 10^2^, respectively. These values remained at baseline in the PBS group. While antibody levels against nucleocapsid protein stayed at baseline, both nucleocapsid- and spike-specific T-cell responses were detected in PBMCs from ORFV-S/N vaccinated NHPs at day 35 by ELISpot (Fig. 4E). Spike-specific responses resulted in a mean of 541 IFN-γ spot forming units (SFU), which were considerably higher as compared to responses against nucleocapsid (mean of 69 SFU).

After challenge with 10^5^ TCID_50_ SARS-CoV-2, half of the vaccinated animals showed detectable viral titers in nose and throat (3/6), and all cleared the virus already at day 4 in contrast to the PBS control group that remained viremic for at least 1 week (Fig. 4F, G). Viral loads in CoVsg PCR-positive primates were 10–100 times lower than in the macaques that received PBS (control). None of the vaccinated NHPs showed the presence of viral titers in BAL, in contrast to all animals in the control group (Fig. 4H).

To evaluate the duration of ORFV-mediated vaccine protection, NHPs were first vaccinated with the ORFV-S/N at a dose of 10^6^ or 3 × 10^7^ PFU or PBS as a control with a 4-week interval, and then challenged with 10^5^ TCID_50_ of SARS-CoV-2 seventeen weeks after the last vaccination (Supplementary Fig. 14). In this long-term experiment, vaccination with 3 × 10^7^ PFU of ORFV-S/N resulted in a significantly improved lung protection as compared to control animals (Supplementary Fig. 14H). Here, 80% of NHPs immunized with 3 × 10^7^ PFU of ORFV-S/N maintained protection against SARS-CoV-2 titers in the lungs.

## Discussion

The emergence of new SARS-CoV-2 variants^1^, the waning antibody levels^47–52^ and decreasing protection over time^2^ demand further development of innovative vaccine technologies. Broadening SARS-CoV-2-specific humoral and cellular immunity by incorporating multiple viral antigens into vaccines, or the use of heterologous vaccination approaches are promising strategies that can improve vaccine-induced immune responses and avoid potential challenges with antigenic imprinting with mutated SARS-CoV-2 VoC^53^. In this context, the highly conserved nucleocapsid protein, in addition to the spike protein, holds promise as an attractive antigen that can provide durable and broadly reactive T cells, thereby enhancing protection against VoC^8,13,17^.

To investigate such a multiantigenic vaccination approach against COVID-19, a novel versatile parapoxviral vector platform based on the attenuated Orf virus strain ORFV D1701-VrV^27,35,42^ was used to develop vaccine candidates expressing the full-length spike protein alone or together with the nucleocapsid protein. The vector vaccine platform presented here provides multiple advantages, particularly compared with recently established viral vector technologies. It can be characterized as a flexible “modular brick system”, allowing the rapid generation and adaptation of vaccine candidates and supports the integration of several transgenes. Due to a short-lived ORFV-specific immunity and an absence of virus-neutralizing antibodies^26,54,55^ effective re-immunization along with the induction of robust and long-lasting immune responses to vector-encoded antigens is feasible^24–31^. Generally, viral vector vaccines are known to provide improved stability and do not require storage at very low temperatures as compared to mRNA vaccines^56^ or special handling^50^, endorsing their use also in low-resource settings.

Our spike antigen design is based on a prefusion-stabilized version to improve vaccine immunogenicity and efficacy^45^. As a result, we observed that both mono- and multiantigenic ORFV-vaccine candidates induced high spike-binding and virus-neutralizing antibody titers already after a single immunization, which further increased after the second administration. Since these elevated titers may have reached a plateau and declined only slightly over a prolonged follow-up period of 150 days, this could explain the limited booster effect seen with the third vaccination. In contrast, the nucleocapsid antigen reflects the full-length, unmodified sequence lacking a signal for membrane export. We speculate that therefore the induction of vaccine-induced antibodies against nucleocapsid was less efficient and thus showed increases after each vaccination with ORFV-S/N. These findings indicate a lack of anti-ORFV immunity impacting vaccine-mediated boosting effects and suggest the feasibility of repeated vaccinations.

Although both spike and nucleocapsid antigens induced specific CD4+ and CD8+ T-cell responses, T-cell frequencies against nucleocapsid were lower than those against spike in our studies. Similar observations were made in subjects vaccinated with the synthetic modified vaccinia Ankara-based S/N vaccine^13^. Speculatively, since nucleocapsid antigen size is smaller and therefore comprises fewer potential CD4+ and CD8+ T-cell epitopes than spike^57^, this may be a reason for the weaker immune responses observed. The addition of an enhanced T-cell stimulation domain facilitating major histocompatibility complex class I and II presentation has been suggested as a promising strategy to amplify T-cell responses^58,59^.

Golden Syrian hamster models show a high genetic similarity to the human ACE2 receptor^60^. Therefore, we investigated vaccine-mediated protection against SARS-CoV-2 challenge in this model. In hamsters, the ORFV-S/N vaccine led to rapid virus clearance in the upper respiratory tract and lungs starting 4 days after the virus challenge and enabling complete protection, whereas the protective effects of the ORFV-S vaccine remained restricted to the lower airways (lungs). Partial protection of the respiratory tract of hamsters after vaccination was also reported for several mono-antigenic spike-targeted vaccines, meanwhile approved or in late stage^61–64^. Our data suggest that preferred immunity conferred by two doses of the multiantigenic ORFV-S/N vaccine may have allowed for improved viral clearance when compared to animals receiving equal doses of ORFV-S. In contrast to the data observed in mice and irrespective of comparable in vitro antigen expression, in a hamster model, the ORFV-S induced higher RBD-specific binding antibody- and virus-neutralizing titers after one and two immunizations as compared to the ORFV-S/N. We hypothesize that this variation might be explained by idiosyncrasies of the animal models used. Importantly, these higher levels were not associated with improved protection after the virus challenge. We speculate that the anti-spike antibody and virus neutralization levels are not the only correlate of protection, at least in this model. Since a correlate of protection against SARS-CoV-2 is not yet established, we are uncertain about how it would be translated to clinical observations. Additional results challenging the notion that neutralizing antibody levels are the sole correlate of vaccine efficacy have also been reported by others^15,23,59^. In this context, those authors speculate that nucleocapsid-mediated immune responses may significantly contribute to full protection^15,23,59^, while it is established that vaccines targeting nucleocapsid alone are ineffective^19^ or provide only poor protection at best^15^.

In spite of an unprecedented body of research and evidence available for SARS-CoV-2^65^, convincing concepts regarding the mechanisms of protection involving nucleocapsid are lacking. Suggested mechanisms include protective cytotoxic T-cell responses^19,66^ as well as natural killer cell-mediated antibody-dependent cellular cytotoxicity^19,67^ as well as Fc-dependent antibody functions^68^ that potentially enhance antigen presentation and cross-priming^66^.

Utilizing a bivalent mRNA-S/N vaccine approach, indications for a relevant involvement of CD8+ T cells in protecting NHPs against SARS-CoV-2 could be obtained^15^. Another line of evidence from humans shows that nucleocapsid-specific T-cell responses are associated with controlling SARS-CoV-2 in the upper airways. Respective responses were present in acutely infected nasopharyngeal tissue during the first week after symptom onset, already before seroconversion and involved gene expression of multiple effector molecules such as IFN-γ^69^. Since it is plausible that antigen-specific T cells can contribute to antiviral effects through eliminating infected cells and thereby preventing virus dissemination, we hypothesize that such effects may have been relevant for the outcomes observed in our study. However, in absence of mechanistic studies our data are insufficient to prove preferential virus clearance at the upper respiratory tract conferred by the multiantigenic vaccine and therefore the presented findings must remain preliminary.

The ORFV-S/N vaccine candidate was also shown to be effective for boosting spike- and nucleocapsid-specific pre-existing memory responses after SARS-CoV-2 infection. Vaccination following a SARS-CoV-2 infection elicited high titers of spike-binding antibodies and potent virus neutralization capacity as well as a more consistent increase of nucleocapsid-binding IgG levels in hamsters, when compared to re-infection. Considering that a large part of the global population meanwhile has been infected with SARS-CoV-2, these findings support the potential of the ORFV-S/N vaccine candidate as an effective booster against COVID-19.

Due to the diminishing relevance of the ancestral virus strain in the course of this project and the ubiquitous emergence of SARS-CoV-2 VoC, the spike design of our multiantigenic vaccine candidate was adapted to the SARS-CoV-2 Beta VoC and investigated for cross-protection against both the ancestral strain and the Delta VoC in hamsters. In challenge experiments with the ancestral strain the modified ORFV-S/N-Beta vaccine conferred full protection comparable to the unmodified ORFV-S/N vaccine. When infecting ORFV-S/N-Beta vaccinated hamsters with SARS-CoV-2 Delta VoC, the complete protection of the lungs was maintained and a reduction of viral loads in the upper airways was achieved as compared to control animals. We believe that these preliminary findings generally support the notion that also this multiantigenic ORFV-S/N vaccine may provide cross-protective activities. In keeping with this, hamsters vaccinated with the synthetic modified vaccinia Ankara-based S/N vaccine COH04S1 targeting the ancestral SARS-CoV-2 strain also showed cross-protection against lower respiratory tract infection and lung pathology when challenged with Omicron BA.1 or BA.2.12.1^70^. Another study comparing mRNA-S vs. mRNA-S+N vaccines in hamsters reported improved protection against SARS-CoV-2 Delta and Omicron VoC when using the multiantigenic vaccine^15^. However, with our study design, we cannot attribute the cross-protection against VoC solely to the addition of the nucleocapsid antigen and the results obtained are inconsistent. The Beta VoC-adapted mono-antigenic spike vaccine has not been tested as a control to exclude the possibility that it is sufficiently cross-protective against both the ancestral strain and the Delta VoC. Further, the cross-protection of ORFV-S/N vaccine against Delta VoC was not evaluated and therefore represents a limitation of this study.

Finally, the multiantigenic ORFV-S/N vaccine candidate was evaluated in the well-established preclinical NHP challenge model, as an important prerequisite for setting up clinical testing in humans. After vaccination with the ORFV-S/N vaccine, rhesus macaques developed humoral immune responses against spike and T cells specific for both the spike- and nucleocapsid protein after two injections. Following SARS-CoV-2 infection, vaccinated animals showed rapid virus clearance in the nose and throat and no detectable virus in bronchoalveolar lavage, suggesting that the SARS-CoV-2 virus remained restricted to the upper airways and did not affect the lungs. The durability of protection against SARS-CoV-2 was further confirmed in a long-term experiment in NHPs. In this study, two doses of 3 × 10^7^ PFU of the ORFV-S/N recombinant afforded lung protection against the SARS-CoV-2 challenge for a period beyond 5 months. This protection was observed in 80% of primates in contrast to none in control animals.

Among the key limitations of the presented study is the lack of conclusive evidence explaining the nucleocapsid-mediated mechanisms of protection in the animal models used. In hamsters, the contribution of nucleocapsid to the observed improved protection in the upper airways including the potential role of mucosal immunity was not clarified and beyond the scope of our study. Since the presented results in NHPs are restricted to the ORFV-S/N vaccine, no valid conclusions could be drawn concerning the value of nucleocapsid in this animal model.

In summary, the presented results highlight the potential of ORFV as a novel vaccine platform. Two multicenter Phase I clinical trials are evaluating the multiantigenic ORFV-S/N lead vaccine candidate (ClinicalTrials.gov identifier: NCT05389319 and NCT05367843) and shall provide additional information on the safety, reactogenicity, immunogenicity and suitability for a heterologous boost approach in humans.

## Supplementary information

Supplemental Information

## Data Availability

All relevant data generated or analyzed during this study are included in this published article and its supplementary information files. Further information and requests for resources and reagents should be directed to the corresponding author, R.A. (ralf.amann@ifiz.uni-tuebingen.de).
