# Supplementary material for: A multiantigenic Orf virus-based vaccine efficiently protects hamsters and nonhuman primates against SARS-CoV-2
Source: NPJ Vaccines. 2024 Oct 16;9:191. doi: 10.1038/s41541-024-00981-2 (PMC11484955; doi:10.1038/s41541-024-00981-2)
Supplement: Supplementary file 1 — Supplemental Information [file 41541_2024_981_MOESM1_ESM.pdf]

Supplementary Information

Supplementary Figure 1

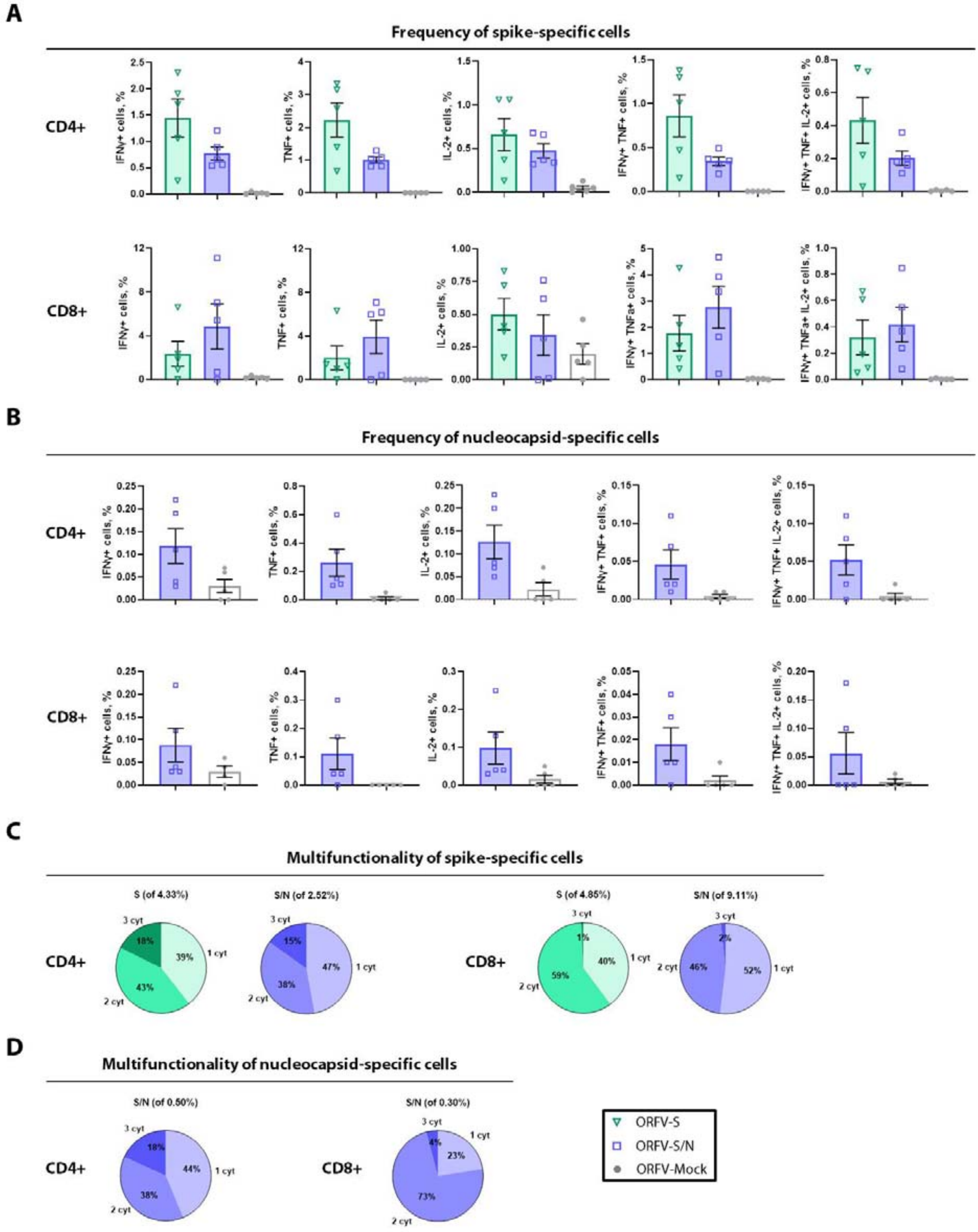

Supplementary Figure 1. Cellular immune responses stimulated by ORFV-S and ORFV-S/N recombinants in mice. CD-1 mice were immunized at day 0 (V1) and 21 (V2) with 10^7 PFU of ORFV-S and ORFV-S/N recombinants, or ORFV-Mock. Frequencies and multifunctionality of A) spike- and B) nucleocapsid-specific CD4+ T cells and CD8+ T cells among splenocytes were assessed at day 28 for cytokine production using an *ex vivo* intracellular cytokine staining assay. In A) and B) bars indicate mean + SEM. IFNγ: Gamma interferon, TNF: Tumor Necrosis Factor, IL-2: Interleukine-2

Supplementary Figure 2

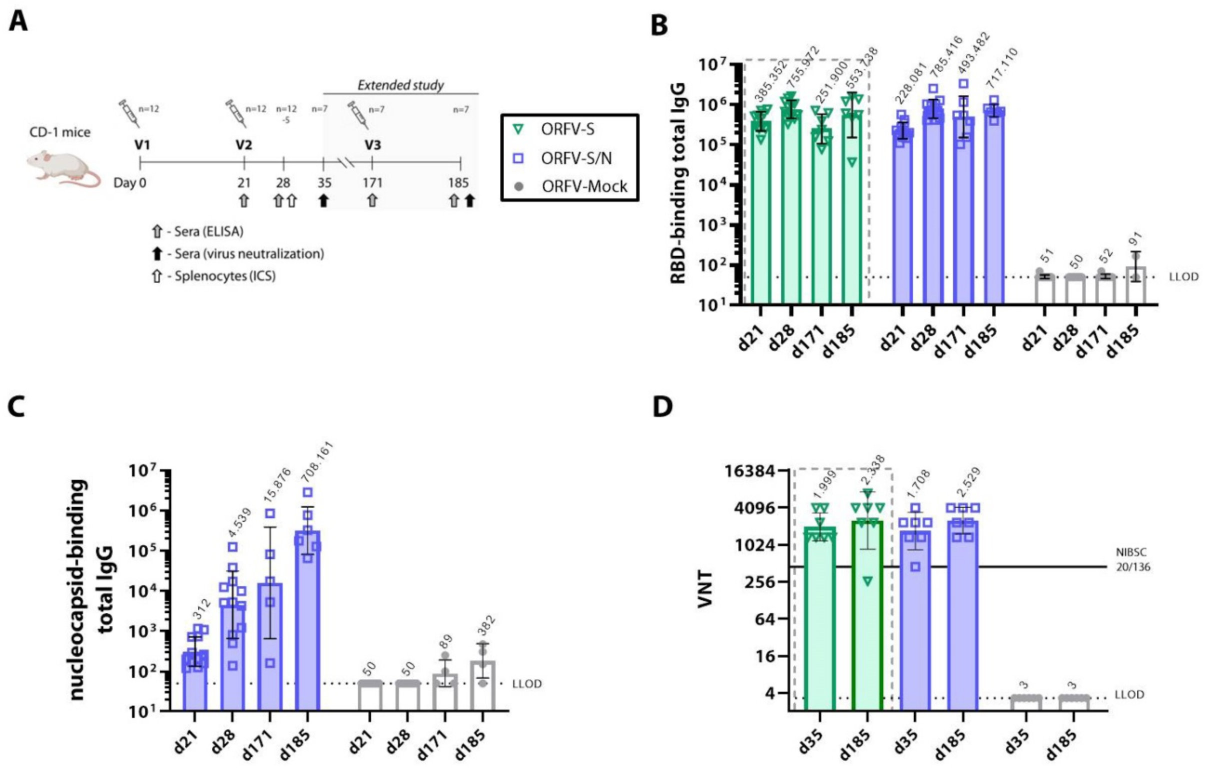

Supplementary Figure 2. Boostability and persistence of antibody responses induced by ORFV-S and ORFV-S/N recombinants. A) CD-1 mice were immunized at day 0 (V1), 21 (V2) and 171 (V3) with 10^7 PFU of ORFV-Mock, or ORFV-S or ORFV-S/N ORFV recombinants. Created in BioRender. Amann, R. (2024) BioRender.com/r59y162. Endpoint titers of B) RBD-specific and C) nucleocapsid-specific total IgG in mouse serum was analyzed at the indicated time points by ELISA. D) VNT of SARS-CoV-2-specific antibodies in serum induced in immunized mice at day 35 and day 185 against SARS-CoV-2 (ancestral strain; Wuhan). The horizontal solid line marks the VNT measured in the WHO standard NIBSC 20/136. In B) – D) data are presented as geometric mean values ± geometric SD. Numbers above the columns denote values of geometric means. The dotted line indicates the LLOD.

Supplementary Figure 3

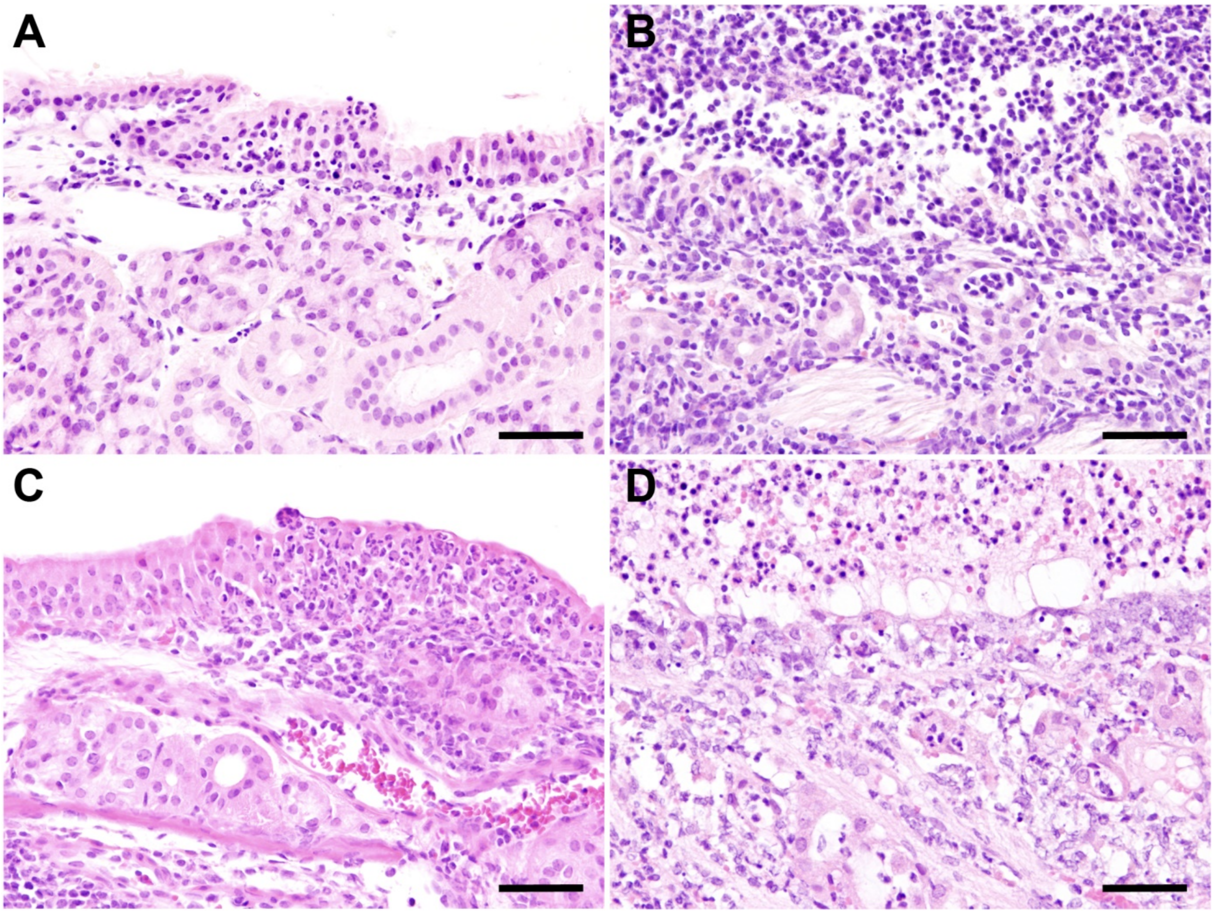

Supplementary Figure 3: Exemplary histopathology findings in nasal mucosa tissue samples of Syrian hamsters four days after SARS-CoV-2 challenge. Rhinitis evidenced in exemplary hamsters A) vaccinated with two doses of ORFV-S/N (10^7 PFU); B) vaccinated with two doses-ORFV-S (10^7 PFU); C) previously infected with SARS-CoV-2 (SARS-CoV-2-recovered) and D) controls (treated with PBS): Note the infiltration of the nasal mucosa predominantly by neutrophils and fewer lymphocytes and macrophages noticed in all animals. Inflammatory cells are found infiltrating the glandular structures and accumulating in the nasal cavity of hamsters vaccinated with B) ORFV-S and D) control animals. Hematoxylin and eosin staining. Bars = 50 µm.

Supplementary Figure 4

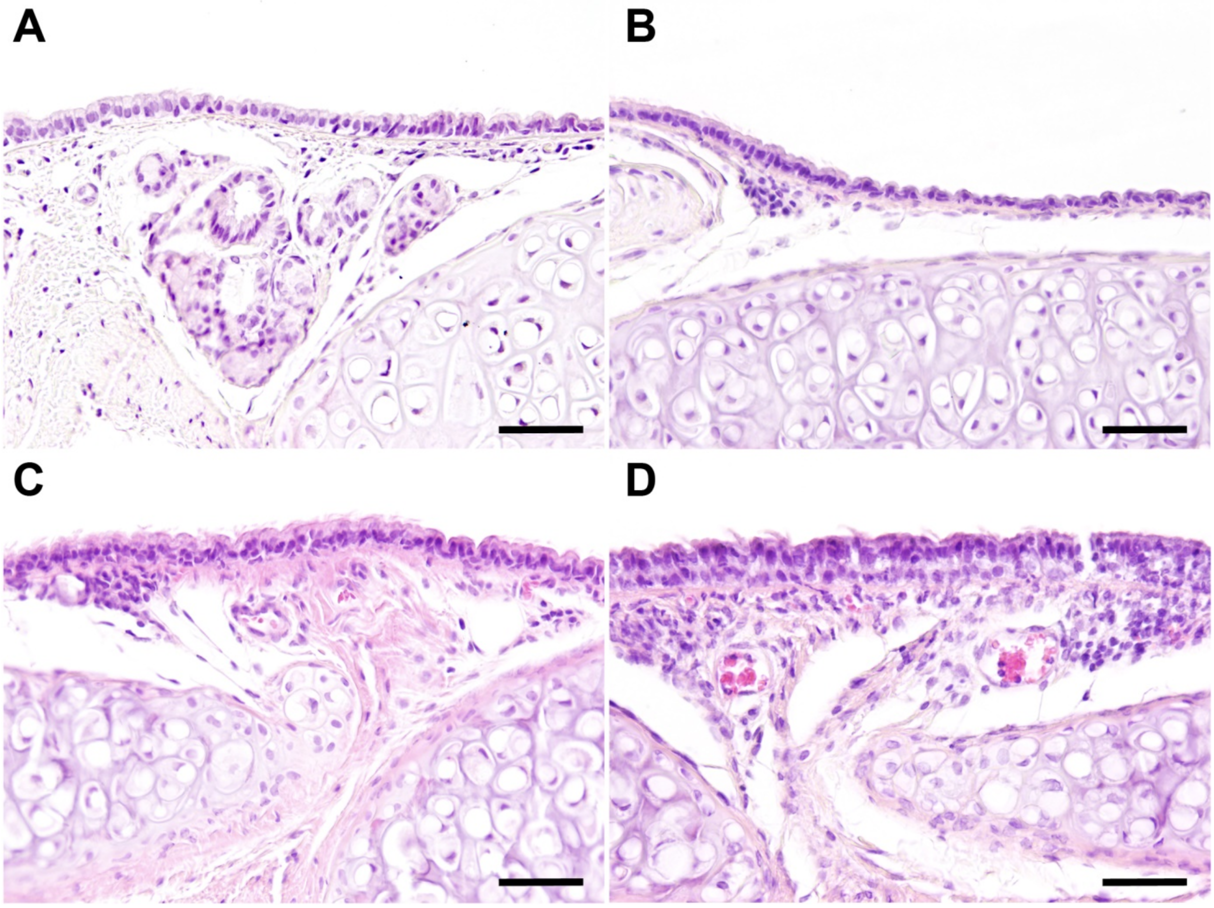

Supplementary Figure 4: Exemplary histopathology findings in tracheal mucosa tissue samples of Syrian hamsters four days after SARS-CoV-2 challenge. Tracheitis evidenced in exemplary hamsters A) vaccinated with two doses of ORFV-S/N (10^7 PFU); B) vaccinated with two doses-ORFV-S (10^7 PFU); C) previously infected with SARS-CoV-2 (SARS-CoV-2 recovered); D) controls (treated with PBS). Note the low to moderate infiltration of the tracheal mucosa by lymphocytes and macrophages in A) ORFV-S/N vaccinated hamsters, appearing more pronounced in the other groups as well as fewer neutrophils in hamsters seen in B), C) and D). Hematoxylin and eosin staining. Bars = 50 µm.

Supplementary Figure 5

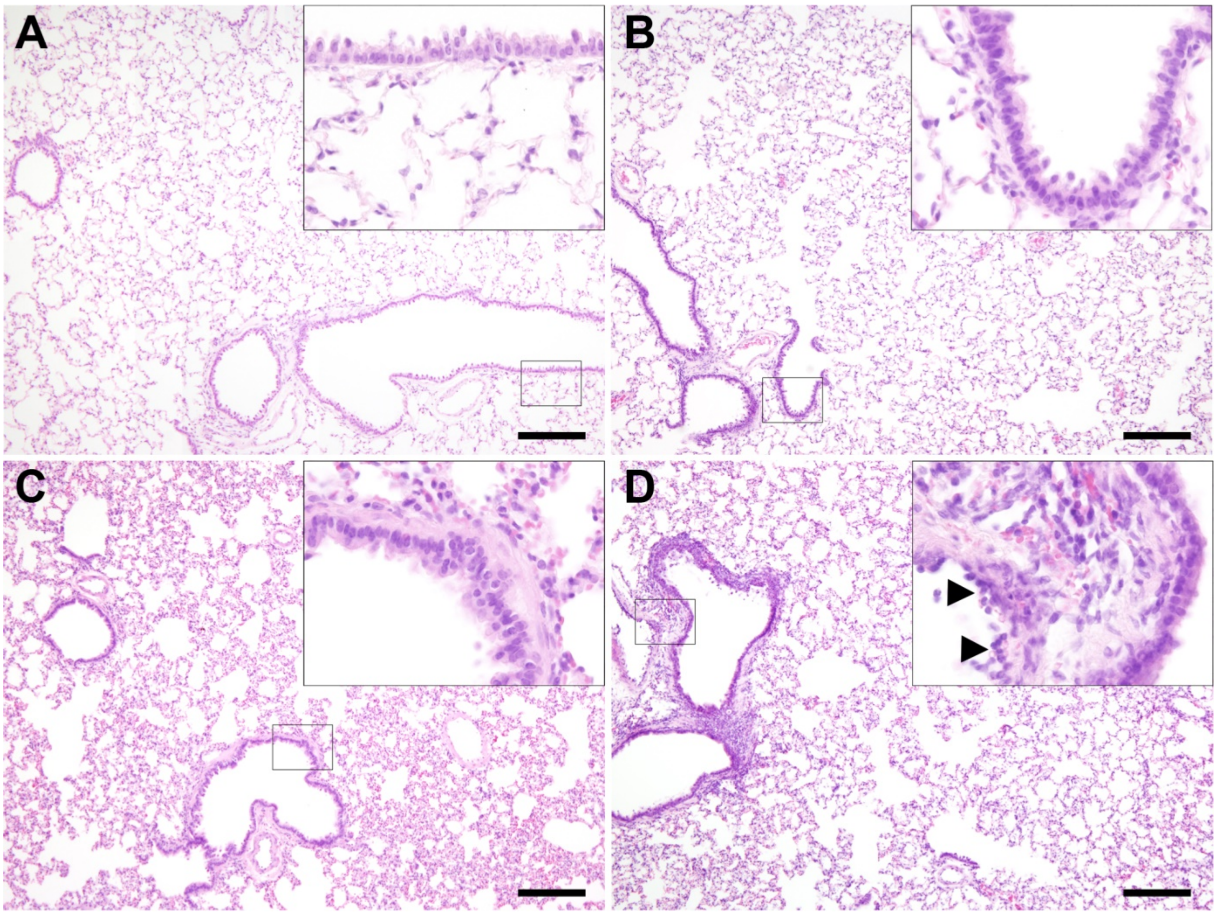

Supplementary Figure 5. Exemplary histopathology findings in lung tissue samples of Syrian hamsters four days after SARS-CoV-2 challenge: perivascular/peribronchial cuffing; bronchiolitis and endothelialitis. Histopathology findings evidenced in lungs of exemplary hamsters A) vaccinated with two doses of ORFV-S/N (10^7 PFU); B) vaccinated with two doses-ORFV-S (10^7 PFU); C) previously infected with SARS-CoV-2 (SARS-CoV-2 recovered); D) controls (treated with PBS). Most of the lung parenchyma lacks histologic lesions at 4 days following SARS-CoV-2 challenge. However, note perivascular/peribronchial cuffing as well as bronchiolitis and endothelialitis (arrowheads) in the hamster of D) control group. Inserts show higher magnifications of the areas indicated by the rectangles. Hematoxylin and eosin staining. Bars = 200 µm.

Supplementary Figure 6

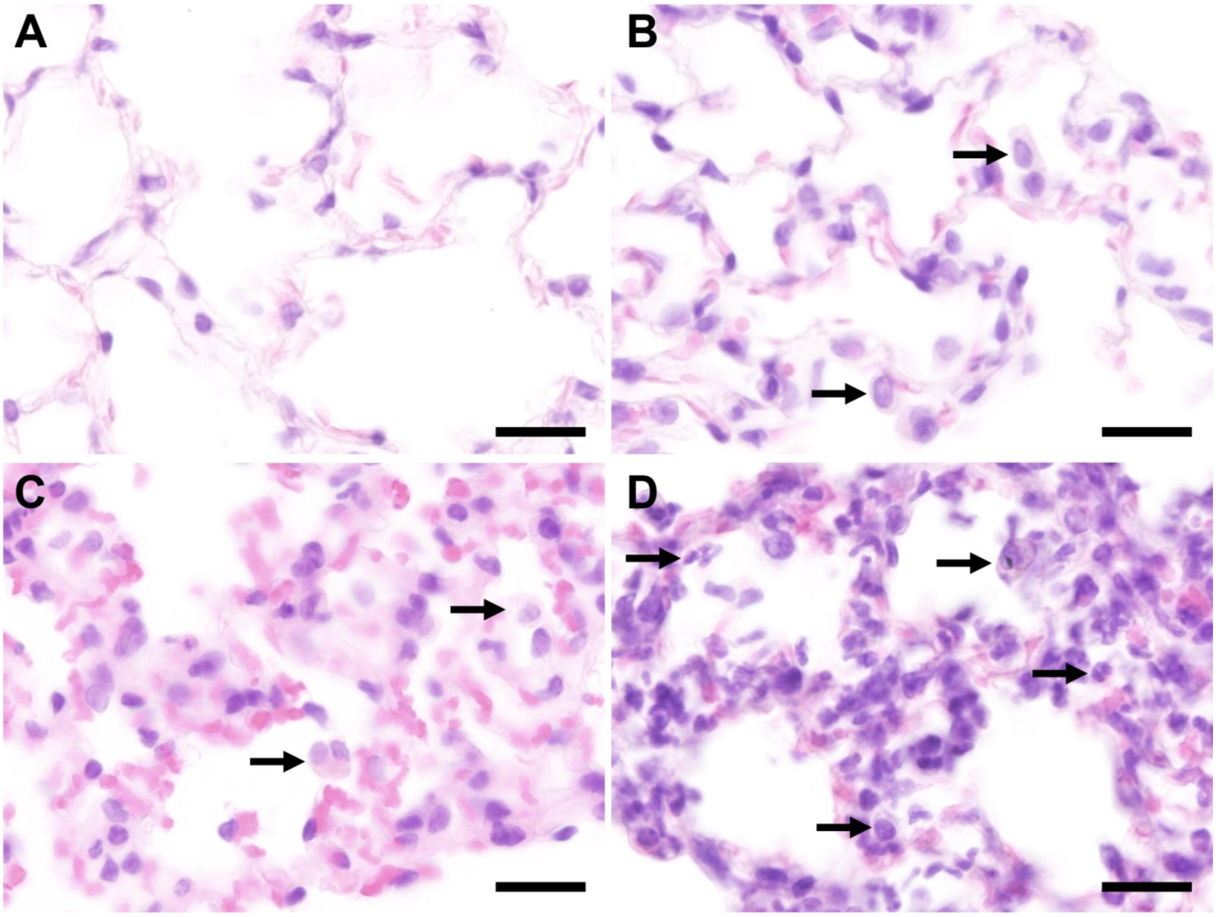

Supplementary Figure 6: Exemplary histopathology findings in lung tissue samples of Syrian hamsters four days after SARS-CoV-2 challenge: alveolitis. Histopathology findings evidenced in lungs of exemplary hamsters: A) vaccinated with two doses of ORFV-S/N (10^7 PFU); B) vaccinated with two doses-ORFV-S (10^7 PFU); C) previously infected with SARS-CoV-2 (SARS-CoV-2 recovered); D) controls (treated with PBS). There is a lack of lesions in the lung parenchyma of hamsters vaccinated with A) ORFV-S/N. Mild alveolitis is found in all other groups i.e. in hamsters vaccinated with B) ORFV-S and C) SARS-CoV-2-recovered animals and D) control animals, characterized by few macrophages and neutrophils infiltrating the alveoli (arrows). Hematoxylin and eosin staining. Bars = 20 µm.

Supplementary Figure 7

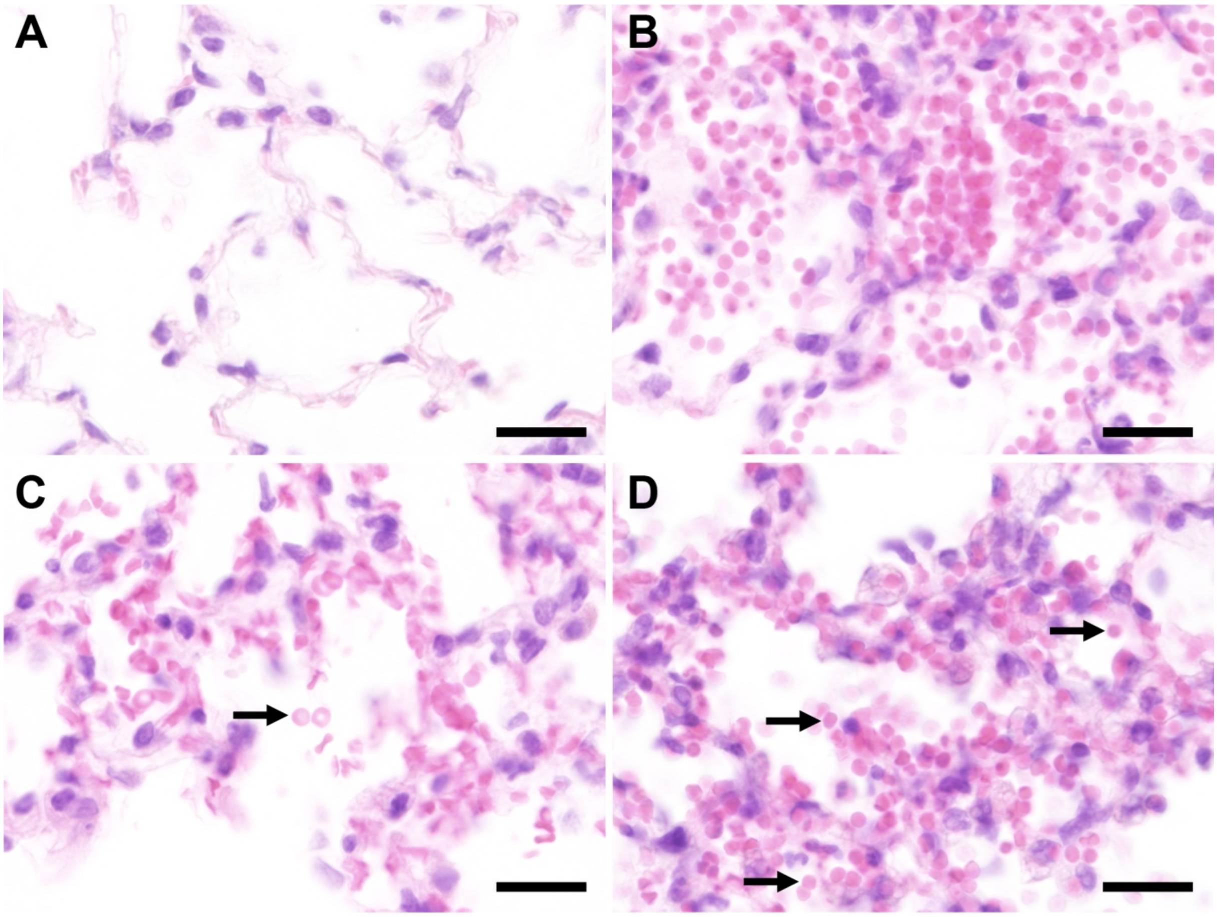

Supplementary Figure 7: Exemplary histopathology findings in lung tissue samples of Syrian hamsters four days after SARS-CoV-2 challenge: hemorrhages. Histopathology findings evidenced in lungs of exemplary hamsters: A) vaccinated with two doses of ORFV-S/N (10^7 PFU); B) vaccinated with two doses-ORFV-S (10^7 PFU); C) previously infected with SARS-CoV-2 (SARS-CoV-2 recovered); D) controls (treated with PBS). Lack of lesions in the lung parenchyma of hamsters vaccinated with A) ORFV-S/N. Focal hemorrhages in hamsters vaccinated with B) ORFV-S, C) SARS-CoV-2-recovered animals and D) control animals characterized by the extravasation of erythrocytes into the alveoli (arrows). Hematoxylin and eosin staining. Bars = 20 µm.

Supplementary Figure 8

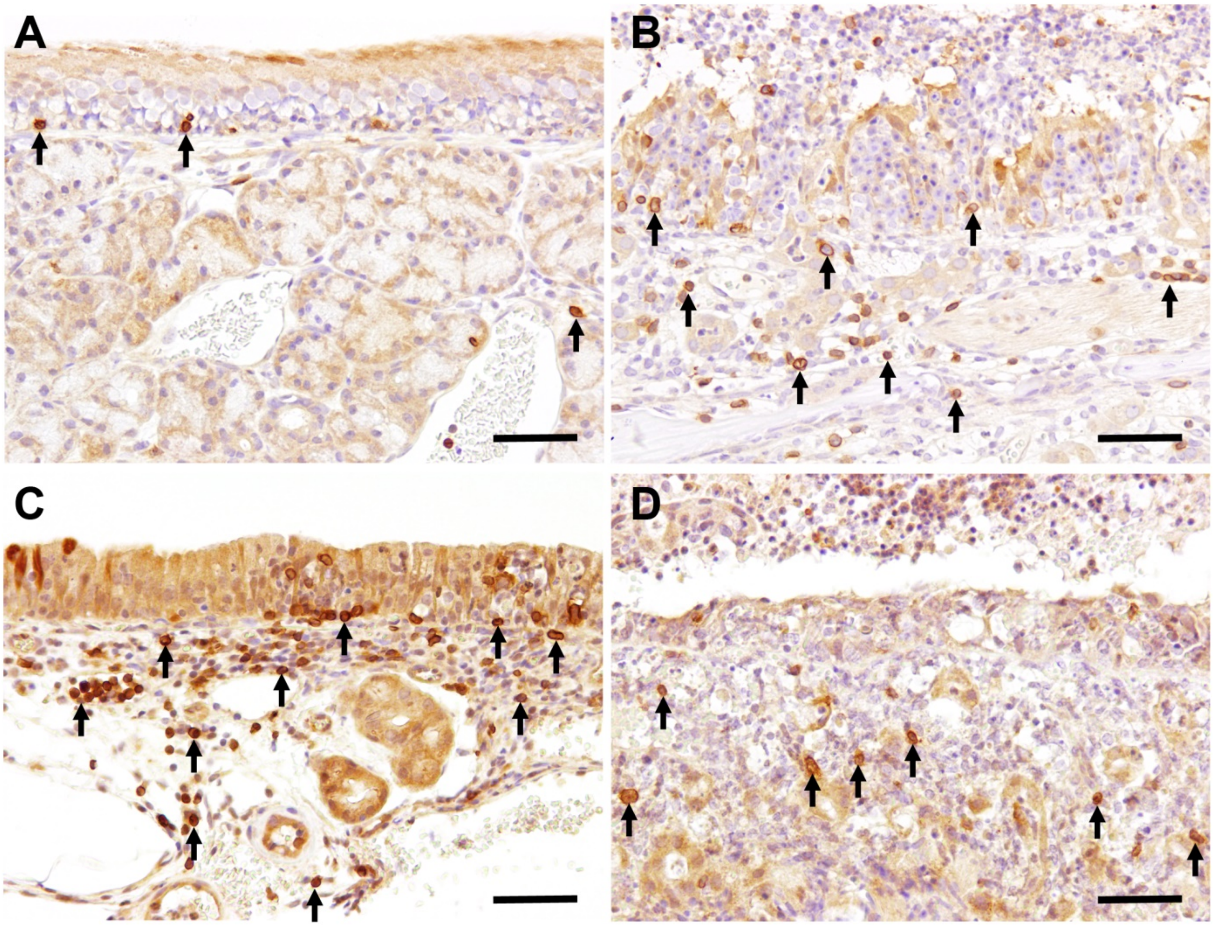

Supplementary Figure 8: Exemplary immunohistochemistry stainings (CD3) in nasal mucosa tissue samples of Syrian hamsters four days after SARS-CoV-2 challenge. Nasal mucosa of hamsters vaccinated with A) two doses of ORFV-S/N (10^7 PFU); B) two doses of ORFV-S (10^7 PFU); C) previously infected with SARS-CoV-2 (SARS-CoV-2 recovered); D) control animals (PBS) four days after SARS-CoV-2 challenge. Note the infiltration of the nasal mucosa by only few CD3+ T cells (arrows) in the hamster vaccinated with A) ORFV-S/N, while higher numbers of CD3+ T cells are present in hamsters vaccinated with B) ORFV-S, C) SARS-CoV-2-recovered and D) PBS controls. Immunohistochemistry for CD3 using the avidin-biotin-peroxidase complex (ABC) method, 3,3'-diaminobenzidine (DAB) as chromogen and Mayer's hematoxylin as counterstaining. Bars = 50 µm.

Supplementary Figure 9

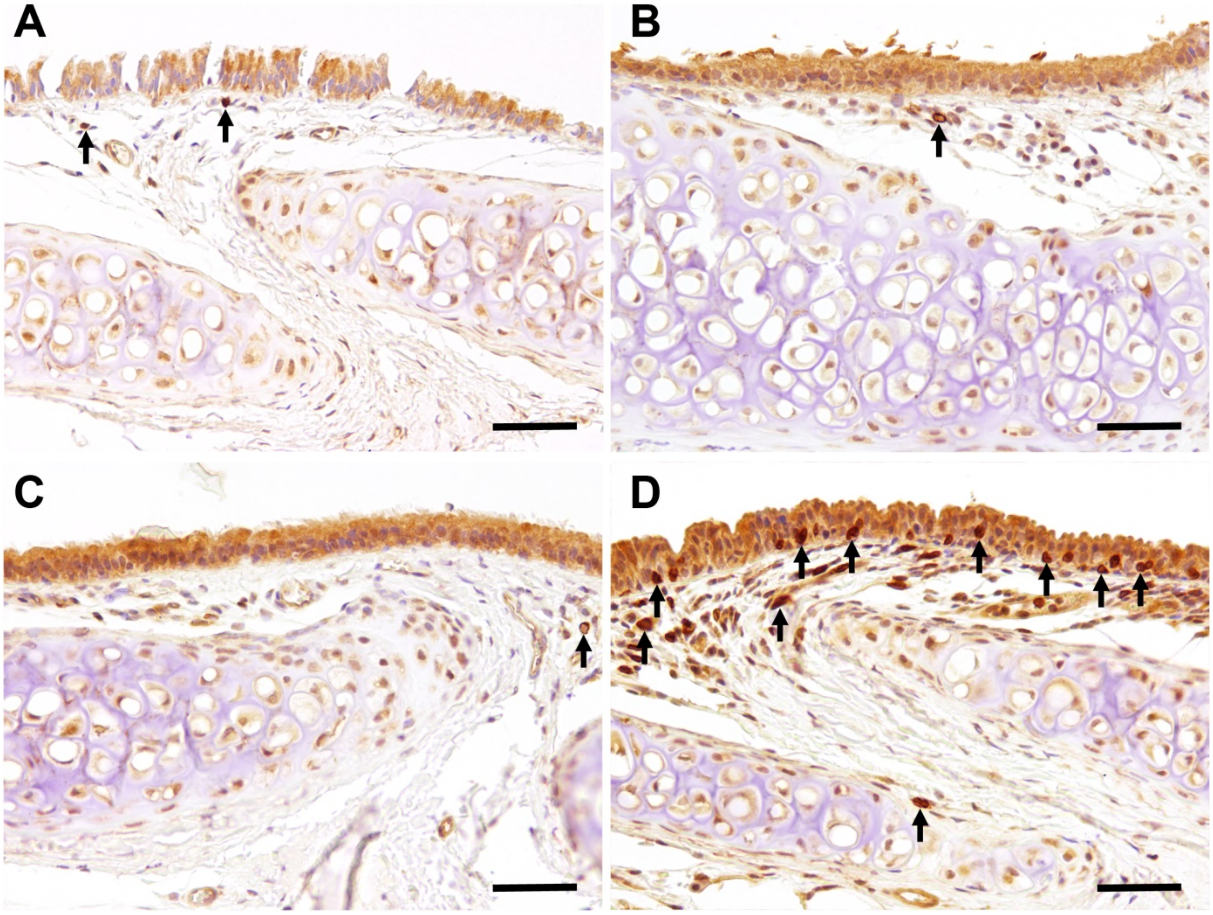

Supplementary Figure 9. Exemplary immunohistochemistry stainings (CD3) in tracheal mucosa tissue samples of Syrian hamsters four days after SARS-CoV-2 challenge. Tracheal mucosa of hamsters vaccinated with A) two doses of ORFV-S/N (10^7 PFU); B) two doses of ORFV-S (10^7 PFU); C) previously infected with SARS-CoV-2 (SARS-CoV-2-recovered); D) control animals (PBS) four days after SARS-CoV-2 challenge. Note the infiltration of the respiratory mucosa by few CD3+ T cells (arrows) in hamsters vaccinated with A) ORFV-S/N, B) ORFV-S and C) SARS-CoV-2-recovered animals, while higher numbers of CD3+ cells are present in the hamster of D) control group. Immunohistochemistry for CD3 using the avidin-biotin-peroxidase complex (ABC) method, 3,3'-diaminobenzidine (DAB) as chromogen and Mayer's hematoxylin as counterstaining. Bars = 50 µm.

Supplementary Figure 10

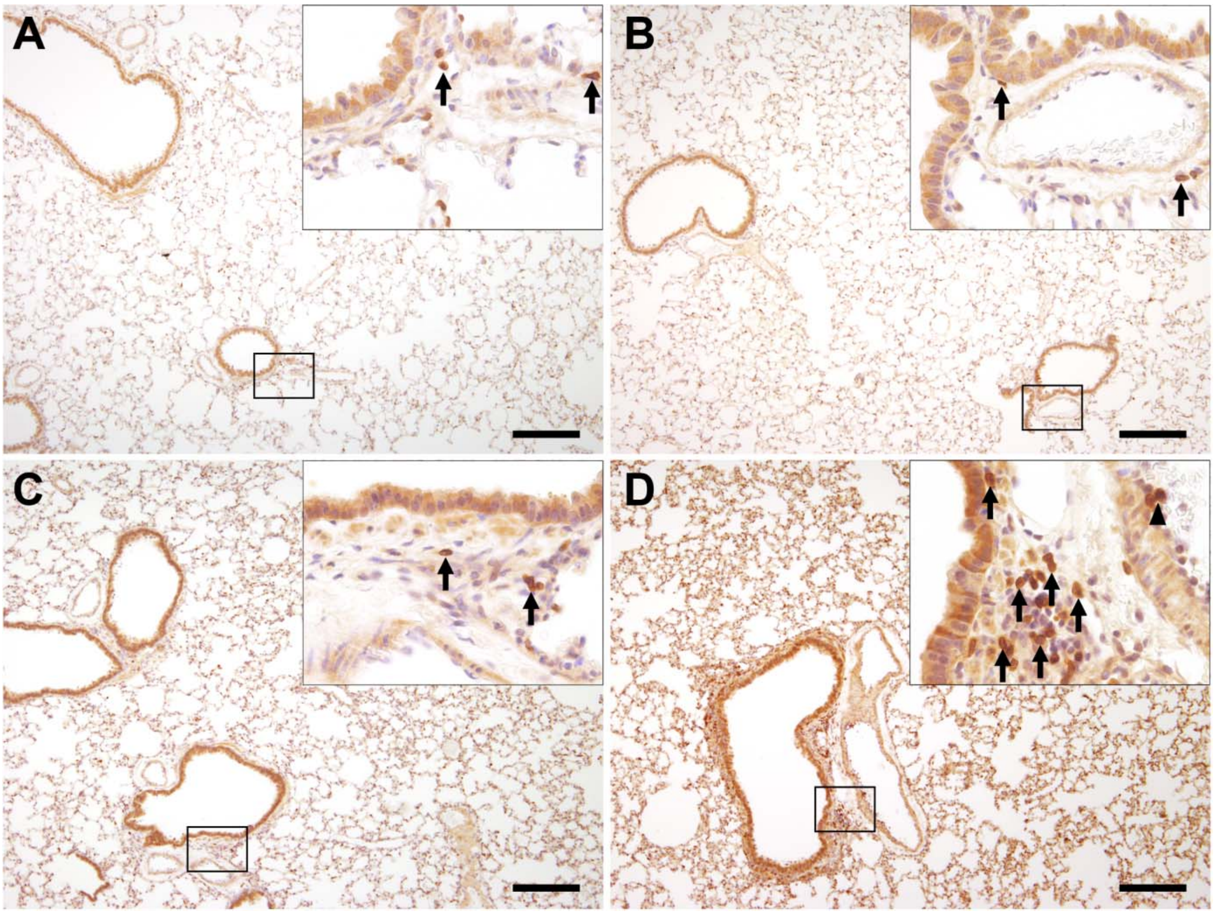

Supplementary Figure 10. Exemplary immunohistochemistry stainings (CD3) in lung tissue samples of Syrian hamsters four days after SARS-CoV-2 challenge. Lung tissue of hamsters vaccinated with A) two doses of ORFV-S/N (10^7 PFU); B) two doses of ORFV-S (10^7 PFU); C) previously infected with SARS-CoV-2 (SARS-CoV-2 recovered); D) control animals (PBS) four days after SARS-CoV-2 challenge. Only few CD3+ T cells (arrows) infiltrate the perivascular/peribronchial tissue of hamsters vaccinated with A) ORFV-S/N or B) ORFV-S and C) SARS-CoV-2-recovered animals, whereas higher numbers of CD3+ T cells are present in the hamster of D) the control group. The latter also show bronchiolitis and endothelialitis (arrowhead). Inserts show higher magnifications of the areas indicated by the rectangles. Immunohistochemistry for CD3 using the avidin-biotin-peroxidase complex (ABC) method, 3,3'-diaminobenzidine (DAB) as chromogen and Mayer's hematoxylin as counterstaining. Bars = 200 µm.

Supplementary Figure 11

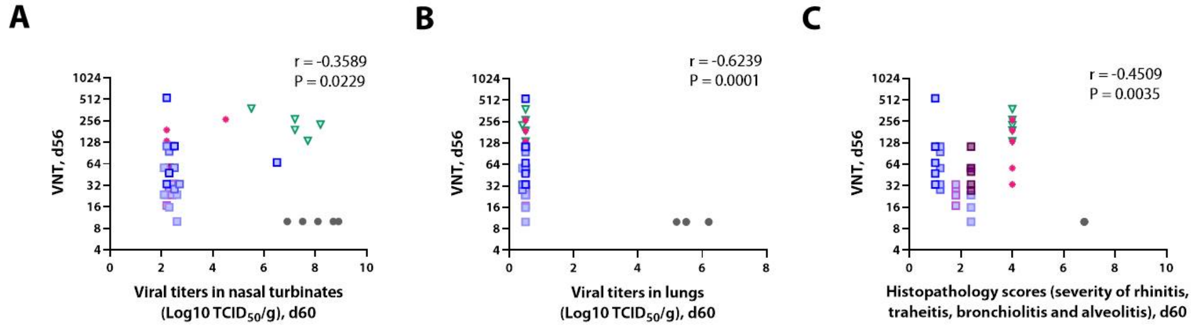

Supplementary Figure 11. Correlation of SARS-CoV-2-specific neutralizing activity induced by ORFV-S and ORFV-S/N vaccination in Syrian hamsters with viral titers and histopathology scores in the respiratory tract after challenge. Spearman's correlation analysis of pre-challenge serum VNT, day 56 with A) viral titers in nose, B) viral titers in lungs and C) histopathological scores four days after challenge, day 60.

Supplementary Figure 12

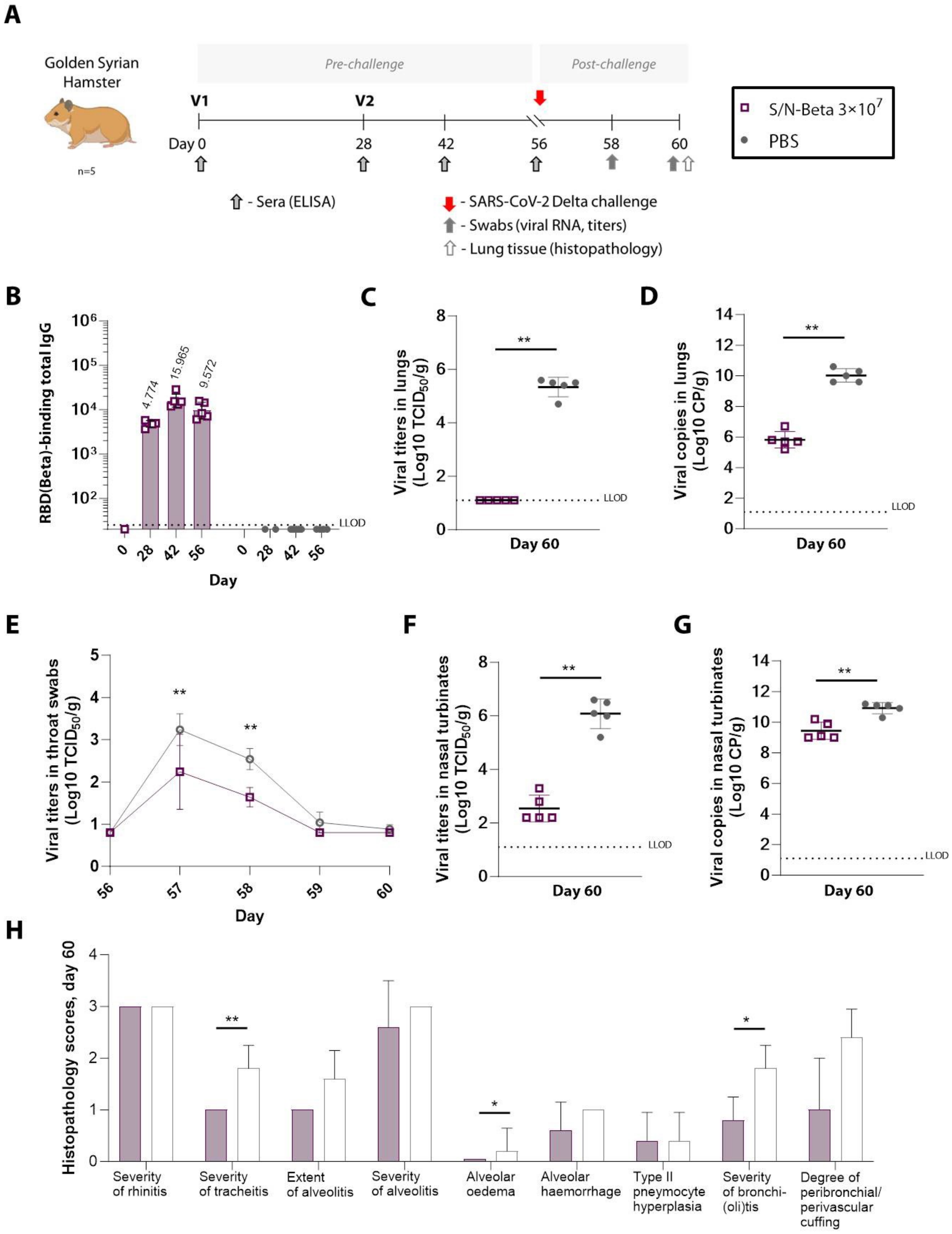

Supplementary Figure 12. Protection against SARS-CoV-2 Delta by vaccination with ORFV-S/N-Beta in Syrian hamsters. A) Hamsters were immunized at days 0 (V1) and 28 (V2) with $3x10^7$ PFU of ORFV-S/N-Beta, a multiantigenic SARS-CoV-2 vaccine candidate containing mutations in the spike protein (K417N, E484K, N501Y) characteristic for the SARS-CoV-2 Beta VoC. PBS was administered as negative control. Animals were challenged with 10^4 $TCID_{50}$ SARS-CoV-2 (Delta strain) at day 56 and monitored for four days. Created in BioRender. Amann, R. (2024) BioRender.com/d60i449. B) Endpoint titers of RBD(Beta)-specific total IgG in serum. In B) data are presented as geometric mean values ± geometric SD. GMT are noted above the columns. Quantification of SARS-CoV-2 Delta C) infectious titers and D) RNA copies in lungs at day 60, E)

infectious titers in throat swabs at days 56-60, and F) infectious titers and G) RNA copies in nasal turbinates at day 60. The dotted line indicates the LLOD. H) Histopathological analysis of tissues at day 60. Scoring was performed according to severity of inspected parameter. In C) – H) data are presented as mean values ± SD. Significance was assessed by Mann-Whitney test. * $p < 0.05$; ** $p < 0.01$; *** $p < 0.001$.

Supplementary Figure 13

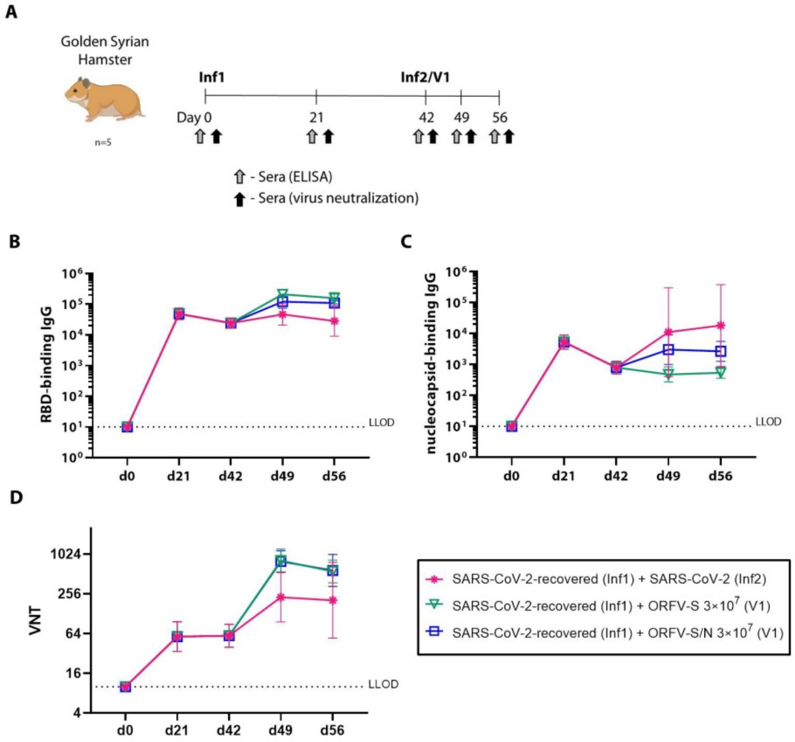

Supplementary Figure 13. Boosting SARS-CoV-2-specific immunity by ORFV-S and ORFV-S/N recombinants in SARS-CoV-2 experienced Syrian hamsters. A) Animals were infected with SARS-CoV-2 (10^2 $TCID_{50}$ intranasally) at day 0 (Inf1) (SARS-CoV-2-recovered) and followed for 56 days. At day 42 hamsters were either vaccinated with $3×10^7$ PFU of ORFV-S or ORFV-S/N recombinants (V1), or challenged/ re-challenged with SARS-CoV-2 (Inf2). Created in BioRender. Amann, R. (2024) BioRender.com/d60i449. B) Endpoint titers of RBD-specific and C) nucleocapsid-specific total IgG in serum were analyzed at the indicated time points by ELISA. D) VNT of SARS-CoV-2-specific antibodies in serum induced in vaccinated hamsters against SARS-CoV-2 (ancestral strain; Wuhan). In B) – D) data are presented as geometric mean values ± geometric SD. Respective GMT are provided in Supplementary Tables 1 - 3. The dotted line indicates the LLOD.

Supplementary Figure 14

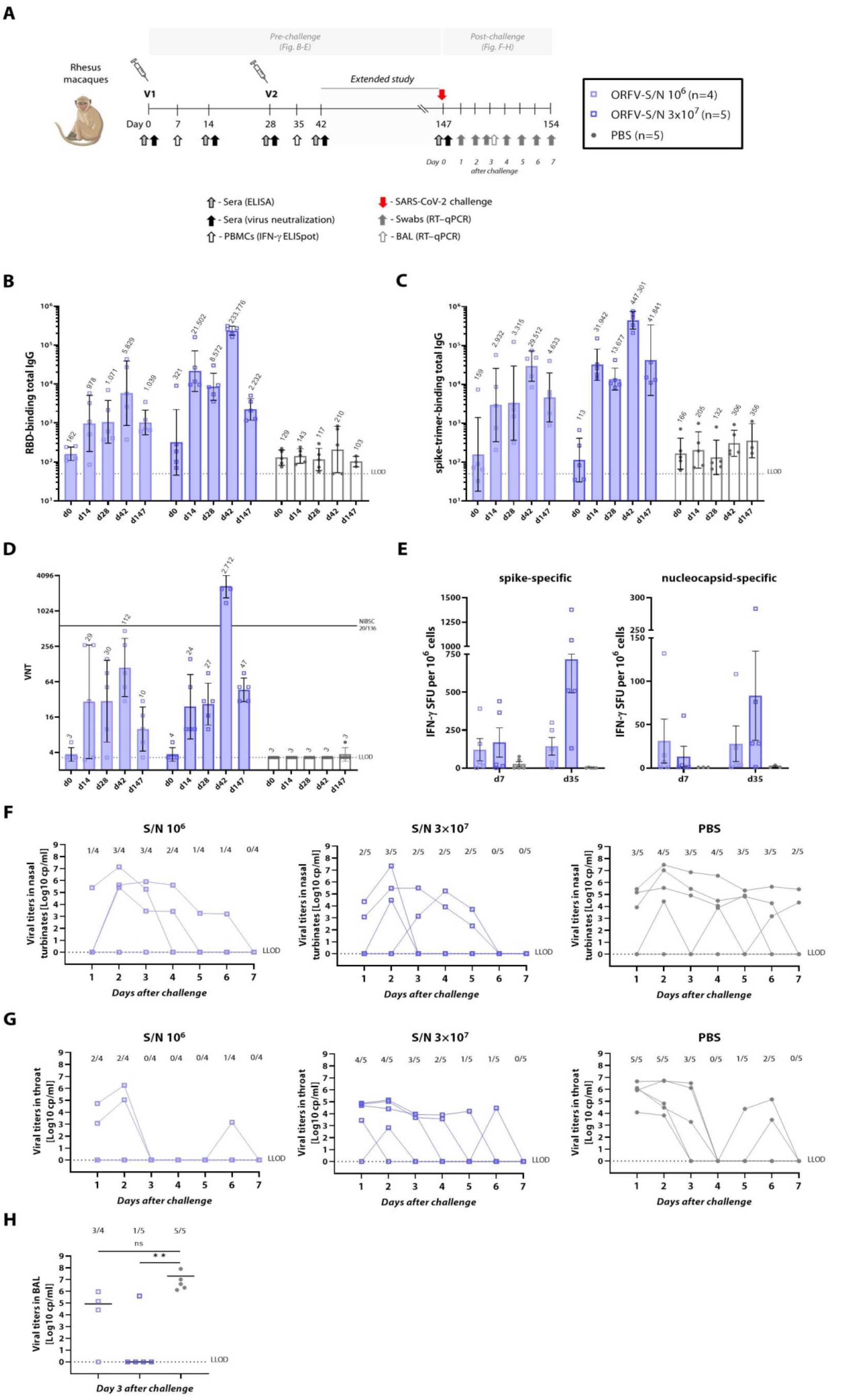

Supplementary Figure 14. Long-term protection induced by ORFV-S/N recombinant-based vaccine in nonhuman primates (NHP). A) NHP were immunized twice with 10^6 or 3×10^7 PFU of ORFV-S/N recombinant, or PBS control at days 0 and 28. All animals were challenged with 10^5 $TCID_{50}$ SARS-CoV-2 administered into nose and trachea at day 147 and monitored for seven days afterwards. Created in BioRender. Amann, R. (2024) BioRender.com/a19n200. Endpoint titers of B) RBD-specific and C) spike-trimer-specific total IgG in serum were analyzed at indicated time points by ELISA. D) VNT of SARS-CoV-2-specific antibodies in serum induced in vaccinated macaques against SARS-CoV-2 (original strain). Horizontal solid line marks the VNT measured in WHO International Reference Panel of anti-SARS-CoV-2 immunoglobulin NIBSC 20/136. In B) – D) data are presented as geometric mean values ± geometric SD. Numbers above the columns denote the GMT. E) IFN-γ-secreting cells in PBMCs at day 7 and 35 after 20 h stimulation with overlapping spike (left graph) and nucleocapsid (right graph) peptide pools, determined by ELISpot. In E) heights of bars indicate mean ± SEM. SARS-CoV-2 viral load in F) nasal turbinate, G) throat swabs and H) bronchoalveolar lavage (BAL) are given at indicated time points after challenge. In F) – H) data are presented as medians. Ratios above bars indicate the number of viral-RNA-positive macaques among all macaques in a group with evaluable samples. The dotted line indicates the LLOD. Significance was assessed by Kruskal-Wallis test. ns, not significant; * $p < 0.05$; ** $p < 0.01$; *** $p < 0.001$.

Supplementary Figure 15

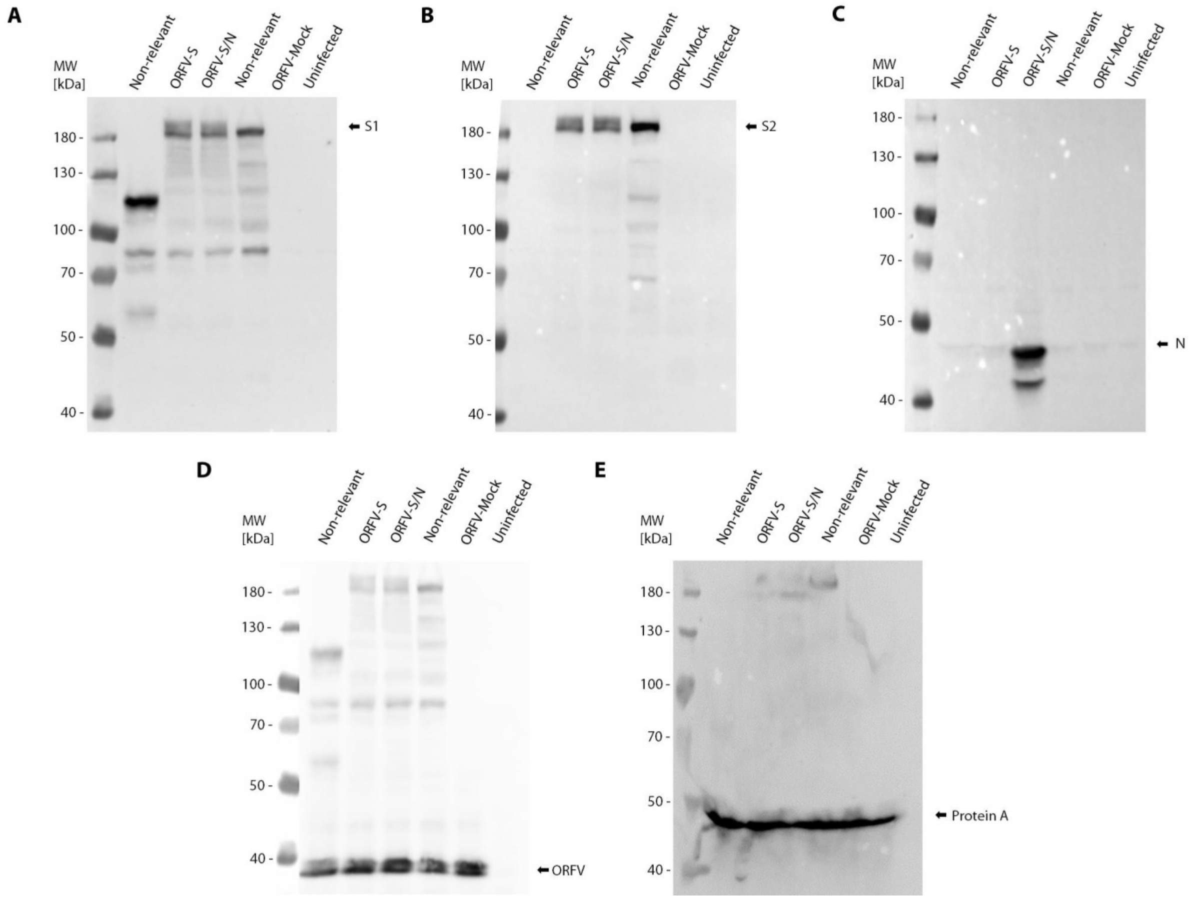

Supplementary Figure 15. Un-cropped and unprocessed images used to generate Fig. 1C, showing transgene expression in ORFV-infected Vero cells. Western Blot analysis of Vero cells infected (MOI 1) for 48 h with the ORFV-S and ORFV-S/N. Antigen expression was evaluated using anti- A) S1, B) S2 and C) N antibodies in cell lysates. D) Anti-ORFV antibodies proved Vero cell infection. E) Loading control antibodies against Protein A were used. Cells infected with ORFV-Mock or uninfected cells were used as controls as indicated. Other bands are non-relevant to this paper.

Supplementary Table 1. Scores* of all assessed histopathological parameters obtained in nasal cavity, trachea and lung in Syrian hamsters four days after SARS-CoV-2 challenge.

Animal Nr.	Group	Extent of alveolitis/ alveolar damage (score 0 to 3)	Severity of alveolitis (score 0 to 3)	Alveolar oedema presence (% slide positive)	Alveolar haemorrhage presence (% slide positive)	Type II pneumocyte hyperplasia presence (% slide positive)	Severity of bronchitis /bronchiolitis (score 0 to 3)	Degree of peribronchial/ perivascular cuffing (score 0 to 3)	Severity of tracheitis (score 0 to 3)	Severity of rhinitis (score 0 to 3)
1	2x ORFV-S/N 1×10^6	0	0	0	0	0	0	0	1	1
2		0	0	0	0	0	0	0	1	2
3		1	1	0	0	0	0	0	1	1
4		0	0	0	0	0	0	0	1	1
5		1	1	0	0	0	0	0	0	1
**n affected animals / group**		**2/5**	**2/5**	**0/5**	**0/5**	**0/5**	**0/5**	**0/5**	**4/5**	**5/5**
**averaged score /group**		**0.4**	**0.4**	**0**	**0**	**0**	**0**	**0**	**0.8**	**1.2**
1	2x ORFV-S/N 1×10^7	0	0	0	0	0	0	0	0	1
2		0	0	0	0	0	0	0	0	1
3		0	0	0	0	0	0	0	0	1
4		0	0	0	0	0	0	0	1	1
5		0	0	0	0	0	0	0	0	1
**n affected animals / group**		**0/5**	**0/5**	**0/5**	**0/5**	**0/5**	**0/5**	**0/5**	**1/5**	**5/5**
**averaged score /group**		**0**	**0**	**0**	**0**	**0**	**0**	**0**	**0.2**	**1.0**
1	2x ORFV-S/N-Beta 1×10^7	0	0	0	0	0	0	1	1	1
2		0	0	0	0	0	0	1	1	1
3		1	1	0	0	0	0	0	1	1
4		1	1	0	0	0	0	0	1	1
5		1	1	0	0	0	0	0	0	1
**n affected animals / group**		**3/5**	**3/5**	**0/5**	**0/5**	**0/5**	**0/5**	**2/5**	**4/5**	**5/5**
**averaged score /group**		**0.6**	**0.6**	**0**	**0**	**0**	**0**	**0.4**	**0.8**	**1.0**
1	2x ORFV-S 1×10^7	1	1	0	1	0	0	0	1	3
2		1	1	0	0	0	0	0	1	3
3		0	0	0	0	0	0	0	1	3
4		0	0	0	0	0	0	0	0	2
5		0	0	0	0	0	0	0	1	3
**n affected animals / group**		**2/5**	**2/5**	**0/5**	**1/5**	**0/5**	**0/5**	**0/5**	**4/5**	**5/5**
**averaged score /group**		**0.4**	**0.4**	**0**	**20**	**0**	**0**	**0**	**0.8**	**2.8**
1	2x ORFV-S/N 1×10^8	1	1	0	0	0	0	0	0	0
2		0	0	0	0	0	0	0	0	1
3		0	0	0	0	0	0	0	0	1
4		0	0	0	0	0	0	0	0	1
5		0	0	0	0	0	0	0	0	1
**n affected animals / group**		**1/5**	**1/5**	**0/5**	**0/5**	**0/5**	**0/5**	**0/5**	**0/5**	**4/5**
**averaged score /group**		**0.2**	**0.2**	**0**	**0**	**0**	**0**	**0**	**0**	**0.8**
1	1x ORFV-S/N 1×10^8	0	0	0	0	0	0	0	1	1
2		0	0	0	1	0	0	0	0	1
3		0	0	0	0	0	0	0	0	1
4		1	1	0	0	0	0	0	1	1
5		0	0	0	1	0	0	0	1	1
**n affected animals / group**		**1/5**	**1/5**	**0/5**	**2/5**	**0/5**	**0/5**	**0/5**	**3/5**	**5/5**
**averaged score /group**		**0.2**	**0.2**	**0**	**40**	**0**	**0**	**0**	**0.6**	**1.0**
1	SARS-CoV-2 recovered	1	1	0	1	0	0	0	1	2
2		1	1	0	0	0	0	0	1	1
3		1	1	0	0	0	0	0	1	3
4		1	1	0	0	0	0	0	1	3
5		1	1	0	0	0	0	0	1	1
**n affected animals / group**		**5/5**	**5/5**	**0/5**	**1/5**	**0/5**	**0/5**	**0/5**	**5/5**	**5/5**
**averaged score /group**		**1.0**	**1.0**	**0**	**20**	**0**	**0**	**0**	**1.0**	**2.0**
1	PBS	1	1	0	1	0	0	0	3	3
2		1	1	0	0	0	0	0	2	3
3		1	1	0	0	0	0	1	1	3
4		1	2	0	1	0	2	2	2	3
5		1	1	0	1	0	1	1	2	3
**n affected animals / group**		**5/5**	**5/5**	**0/5**	**3/5**	**0/5**	**2/5**	**3/5**	**5/5**	**5/5**
**averaged score /group**		**1.0**	**1.2**	**0**	**40**	**0**	**0.6**	**0.8**	**2.0**	**3.0**

*Explanation of scores of microscopic examination. Severity of alveolitis, bronchitis, tracheitis, rhinitis: 0 = no inflammatory cells, 1 = few inflammatory cells, 2 = moderate number of inflammatory cells, 3 = many inflammatory cells. Extent of alveolitis/alveolar damage per total area of lung tissue: 0 = 0%, 1 = <25%, 2 = 25 - 50%, 3 = >50%. Presence of alveolar oedema, alveolar haemorrhage, type II pneumocyte hyperplasia: 0 = no, 1 = yes. Degree of peribronchial/perivascular cuffing: 0 = none, 1 = 1 - 2 cells, 2 = 3 - 10 cells, 3 = >10 cells.

Supplementary Table 2. Survival and scores* of macroscopic examination of pulmonary alterations in Syrian hamsters four days after SARS-CoV-2 challenge.

Animal Nr.	Group	Survival to the end of experiment (% of animals)	Affected lung* (% range of affected lung area)
1	2x ORFV-S/N 1×10^6	100	0
2		100	0
3		100	0
4		100	0
5		100	0
**% of affected animals / group**		**0**	**0**
**averaged % of affected lung /group**			**0**
1	2x ORFV-S/N 1×10^7	100	0
2		100	5
3		100	0
4		100	0
5		100	0
**% of affected animals / group**		**0**	**20**
**averaged % of affected lung /group**			**1**
1	2x ORFV-S/N-Beta 1×10^7	100	0
2		100	0
3		100	0
4		100	0
5		100	0
**% of affected animals / group**		**0**	**0**
**averaged % of affected lung /group**			**0**
1	2x ORFV-S 1×10^7	100	10
2		100	90
3		100	10
4		100	20
5		100	90
**% of affected animals / group**		**0**	**100**
**averaged % of affected lung /group**			**44**
1	2x ORFV-S/N 1×10^8	100	0
2		100	0
3		100	0
4		100	0
5		100	0
**% of affected animals / group**		**0**	**0**
**averaged % of affected lung /group**			**0**
1	1x ORFV-S/N 1×10^8	100	0
2		100	0
3		100	0
4		100	0
5		100	0
**% of affected animals / group**		**0**	**0**
**averaged % of affected lung /group**			**0**
1	SARS-CoV-2 recovered	100	50
2		100	20
3		100	55
4		100	70
5		100	0
**% of affected animals / group**		**0**	**80**
**averaged % of affected lung /group**			**39**
1	PBS	100	5
2		100	20
3		100	15
4		100	25
5		100	50
**% of affected animals / group**		**0**	**100**
**averaged % of affected lung /group**			**23**

*Explanation of scores of macroscopic examination. Percentage range of affected lung area: 0% = undetectable, 0 - 20% = slight, 20 - 40% = mild, 40 - 60% = moderate, 60 - 80% = marked, 80 - 100% = severe, 100% = diffuse/complete

Supplementary Table 3. RBD-specific antibody titers in SARS-CoV-2 experienced Syrian hamsters.

Group	S1RBD-specific IgG geometric mean endpoint titer				
	d0	d21	d42	d49	d56
SARS-CoV-2 (d0) + SARS-CoV-2 (d42)	10	54.902	26.904	46.607	28.263
SARS-CoV-2 (d0) + ORFV-S 3×10^7 (d42)	10	52.412	23.982	210.640	157.244
SARS-CoV-2 (d0) + ORFV-S/N 3×10^7 (d42)	10	40.541	21.925	120.413	108.717

Supplementary Table 4. Nucleocapsid-specific antibody titers in SARS-CoV-2 experienced Syrian hamsters.

Group	Nucleocapsid-specific IgG geometric mean endpoint titer				
	d0	d21	d42	d49	d56
SARS-CoV-2 (d0) + SARS-CoV-2 (d42)	10	6.209	741	11.129	18.243
SARS-CoV-2 (d0) + ORFV-S 3×10^7 (d42)	10	5.753	833	478	543
SARS-CoV-2 (d0) + ORFV-S/N 3×10^7 (d42)	10	4.225	814	3.043	2.665

Supplementary Table 5. Virus neutralization titers in SARS-CoV-2 experienced Syrian hamsters.

Group	Virus neutralization geometric mean endpoint titer				
	d0	d21	d42	d49	d56
SARS-CoV-2 (d0) + SARS-CoV-2 (d42)	10	68	70	226	204
SARS-CoV-2 (d0) + ORFV-S 3×10^7 (d42)	10	44	44	814	558
SARS-CoV-2 (d0) + ORFV-S/N 3×10^7 (d42)	10	63	67	792	576
